# Natural killer cell homing and trafficking in tissues and tumors: from biology to application

**DOI:** 10.1038/s41392-022-01058-z

**Published:** 2022-06-29

**Authors:** Guang he Ran, Yu qing Lin, Lei Tian, Tao Zhang, Dong mei Yan, Jian hua Yu, You cai Deng

**Affiliations:** 1 Department of Immunology, School of Basic Medical, Jiamusi University, 154007 Jiamusi, China; 2 Institute of Materia Medica, College of Pharmacy, Army Medical University, 400038 Chongqing, China; 3 Department of Hematology and Hematopoietic Cell Transplantation, City of Hope National Medical Center, Los Angeles, CA 91010 USA; 4 Department of Clinical Hematology, College of Pharmacy, Army Medical University, 400038 Chongqing, China

**Keywords:** Tumour immunology, Innate immune cells

## Abstract

Natural killer (NK) cells, a subgroup of innate lymphoid cells, act as the first line of defense against cancer. Although some evidence shows that NK cells can develop in secondary lymphoid tissues, NK cells develop mainly in the bone marrow (BM) and egress into the blood circulation when they mature. They then migrate to and settle down in peripheral tissues, though some special subsets home back into the BM or secondary lymphoid organs. Owing to its success in allogeneic adoptive transfer for cancer treatment and its “off-the-shelf” potential, NK cell-based immunotherapy is attracting increasing attention in the treatment of various cancers. However, insufficient infiltration of adoptively transferred NK cells limits clinical utility, especially for solid tumors. Expansion of NK cells or engineered chimeric antigen receptor (CAR) NK cells ex vivo prior to adoptive transfer by using various cytokines alters the profiles of chemokine receptors, which affects the infiltration of transferred NK cells into tumor tissue. Several factors control NK cell trafficking and homing, including cell-intrinsic factors (e.g., transcriptional factors), cell-extrinsic factors (e.g., integrins, selectins, chemokines and their corresponding receptors, signals induced by cytokines, sphingosine-1-phosphate (S1P), etc.), and the cellular microenvironment. Here, we summarize the profiles and mechanisms of NK cell homing and trafficking at steady state and during tumor development, aiming to improve NK cell-based cancer immunotherapy.

## Introduction

Natural killer (NK) cells are an important part of the innate immune system. Unlike T and B cells, they rapidly attack target cells without prior sensitization. These group I innate lymphoid cells (ILCs) express T-bet and produce T helper cell type 1 (Th1)-associated cytokines, such as interferon-γ (IFN-γ).^1–3^ As they mature, NK cells are able to migrate from the bone marrow (BM) into the blood and settle down in peripheral tissues. Their ability to circulate between lymphatic and non-lymphoid organs results in their presence in most tissues.^4–6^ NK cells also acquire effector functions as they mature, including natural cytotoxicity that target cells such as tumor cells or cells infected with virus.^7^ NK cells also produce cytokines, growth factors, and chemokines that help shape adaptive immune responses by interacting with other immune cells.^7^ These powerful effector functions and extensive tissue distribution enable NK cells to play important roles in a variety of diseases, including cancer, infectious diseases, autoimmunity, and chronic inflammation.^8–11^ NK cells are considered to be an effective innate immune cell subset involved in immune surveillance of hematological malignancies and solid tumors as well as metastatic spreading.^12–14^ Indeed, infiltration of cytotoxic NK cells into tumors is a positive prognostic marker for a variety of cancers, including melanoma, renal cell carcinoma (RCC), liver cancer, lung, and breast cancer.^15–19^

**Fig. 1:**
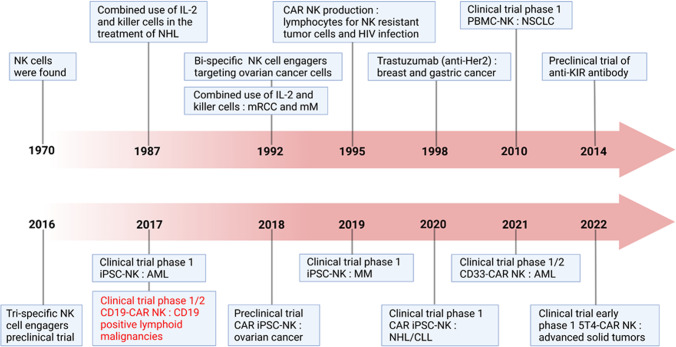
The development of immunotherapy based on NK cells. Applications of various NK cell strategies in preclinical and clinical trials are listed. Since the discovery of NK cells in 1970s, NK cells have been used in clinical treatment. The combination of IL-2 and killer cells in the treatment of non-Hodgkin’s lymphoma (NHL),^302^ metastatic RCC (mRCC),^303^ and metastatic melanoma (mM), starting from 1987. Antibodies enhancing NK cell effector function were produced starting from 1992, including NK bi-specific monoclonal antibody targeting ovarian cancer cells in 1992,^304^ Trastuzumab (anti-Her2) in the treatment of breast cancer and gastric cancer, in 1998, anti-KIR antibody preclinical test in 2014,^305^ and tri-specific NK cell engagers preclinical test in 2016.^306^ Clinical trials using ex vivo expanded peripheral blood mononuclear cell (PBMC)-derived NK cells for non-small cell lung cancer (NSCLC) were started in 2010,^307^ while iPSC-derived NK (iPSC-NK) cells were used for AML treatment in 2017,^308^ and for multiple myeloma (MM) in 2019.^309^ CD4 zeta chimeric receptors were firstly introduced into NK cells and showed highly effective at killing target cells expressing HIV gp120 in vitro in 1995.^22^ Recently, a variety of clinical phase I or II trials were started, including Phase1/2 CD19-chimeric antigen NK cells for CD19-positive lymphoid malignancies in 2017 (NCT03056339), Phase1/2 CD33-CAR NK cells for AML treatment in 2021,^310^ and 5T4-CAR NK cells for treating advanced solid tumors (NCT05194709) in 2022. Preclinical trial for CAR iPSC-NK cells for ovarian cancer were started in 2018,^26^ while clinical trials of CAR iPSC-NK cells for treating NHL or chronic lymphoblastic leukemia (CLL) in 2020.^28^ Created with BioRender.com

NK cell-based immunotherapy has been explored in clinical trials since the 1980s through adoptive transfer of autologous lymphokine-activated killer cells into patients with advanced cancers, who have shown marked tumor regression.^20,21^ The CD4 zeta chimeric receptor was the first chimeric antigen receptor (CAR) introduced to NK cells, and it was highly effective at killing target cells expressing HIV gp120 in vitro.^22^ In 2002, Ruggeri et al. reported that outcomes of patients with acute myeloid leukemia (AML) transplanted with hematopoietic stem cells (HSCs) correlated positively with the anti-leukemic effect of allogeneic NK cells.^23^ In a pioneering study in 2005, Miller et al. adoptively transferred allogeneic activated NK cells into patients with AML, inducing major anti-tumor responses.^24^ The first human clinical trial, reported in 2018, tested the safety of transferring CAR NK-92 cells into patients with relapsed and refractory AML, and it targeted CD33.^25^ In the same year, the Kaufman group first derived CAR NK cells from induced pluripotent stem cells (iPSCs) expressing a CAR. In animal models, those cells appeared to have better cytotoxic activity against solid tumors than peripheral blood-derived CAR NK cells or CAR T cells.^26,27^ In 2020, a clinical trial (NCT03056339) derived HLA-mismatched NK cells from umbilical cord blood and armed them with CD19-CAR showed significant benefits in relapsed or refractory CD19-positive lymphoma and leukemia, without substantial toxicity.^28^ This suggests that CAR NK cells have the potential to become effective allogeneic anti-cancer immunotherapeutic products, which have sparked great interests in the field of cancer immunotherapy. However, the therapeutic efficiency of nonengineered NK cells is much less than desired, especially in solid tumors.^8,12,29^ The development of NK cell immunotherapy is summarized in Fig. 1.

The tumor microenvironment (TME) contains a multitude of immunosuppressive factors for NK cells.^30–32^ The density of endogenous NK cells is extremely low in various human solid tumors, with no more than 100 cells per mm^2^ in contrast to several hundred CD8^+^ T cells per mm^2^.^33^ This suggests that understanding the trafficking of NK cells into the TME may spawn better strategies for improving the effectiveness of NK cell-based immunotherapy for cancer patients. Here, we review the latest data on the trafficking and homing of NK cells into normal tissues and tumors, focusing mainly on the BM, the blood circulation, tissue specificity, intra-tumor movement, and factors controlling NK cell trafficking.

## NK cell development, maturation, and activation

NK cells, especially in mice, were thought to differentiate from HSCs exclusively in the BM and then migrate to peripheral tissues.^34,35^ Recently, however, NK progenitors and immature cells were reported to also mature in secondary lymphoid tissues, such as lymph nodes (LNs), tonsils, and spleen.^1,6,36^ HSCs differentiate gradually from lymphoid-induced pluripotent progenitor cells and then become common lymphoid progenitor cells (CLPs).^34,37^ CLPs produce the precursors of all lymphocyte populations, including T cells, B cells, NK cells, and recently defined ILC subsets.^1^ Upon differentiation, NK cells show dramatic phenotypic changes, including expression of cytokine receptors (e.g., CD122, CD127), adhesion molecules (e.g., integrins), chemoattractant receptors, and activating or inhibitory receptors that control their maturation, migration, and effector functions.^34,37^

In mice, HSC populations are defined as lineage negative (Lin^−^) stem cell antigen (Sca)^+^ CD117 (c-Kit)^+^, which differentiate into CLP populations (Lin^−^Sca^low^CD117^low^CD135^+^CD127 (IL7Rα)^+^).^38–40^ CLPs then differentiate into NK progenitors (NKP, Lin^−^CD122^+^NK1.1^−^DX5^−^)^35^ through an intermediate stage, defined as pre-NKPs (Lin^−^CD117^−^CD135^−^CD27^+^CD244^+^CD117^lo^CD127^+^CD122^−^) that lack the expression of CD122.^40^ The acquisition of CD122, the common β chain of IL-2 and IL-15, is a critical step in the differentiation of NK cells. IL-15 can promote NK cell differentiation, maturation, and survival, and it is constitutively produced by BM stromal cells, activated monocytes, and dendritic cells (DCs).^41^ Expression of the activation receptor complex NKG2D/DNAX-activating protein of 10 kDa (DAP10) defines the initial stage of NKPs’ progression to immature NK (iNK) cells.

**Fig. 2:**
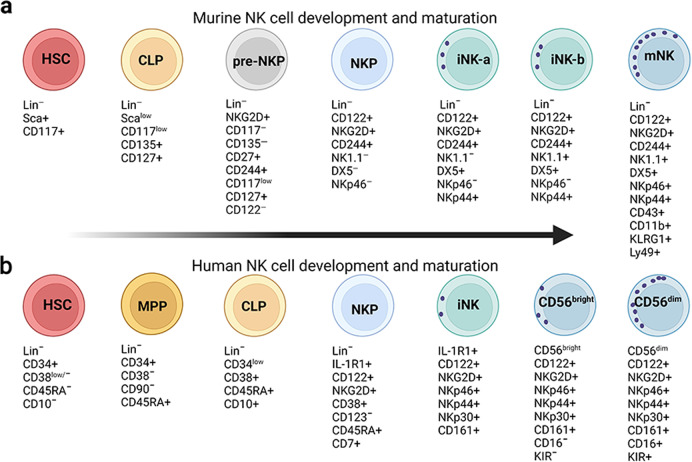
Schematic representation of the cellular intermediates in both murine and human NK cell development. Surface antigens help distinguish the intermediate populations in NK development. **a** Linear path (left to right) in the development of murine NK cells from HSC to mature CD11b^+^CD27^−^DX5^+^ NK cells. **b** Linear path (left to right) in the development of human NK cells from HSC to mature CD56^dim^ NK cells. + (expression), − (no expression), hi (high expression), low (low expression). Created with BioRender.com

Previous studies defined iNK cells and mature NK (mNK) cells as Lin^−^CD122^+^NK1.1^+^DX5^−^ and Lin^−^CD122^+^NK1.1^+^DX5^+^, respectively, and showed that expression of DX5 (also called CD49b or integrin α2) initiates the terminal stage of NK cell maturation.^2^ However, this scheme neglects a subpopulation of NK cells—NK1.1^−^DX5^+^ cells—which account for 10% of Lin^−^CD122^+^ cells. Our recent study refined this developmental process, defining CD122^+^NK1.1^−^DX5^+^NKp46^−^ and CD122^+^NK1.1^+^DX5^+^NKp46^−^ cells as iNK-a and iNK-b populations, respectively, and CD122^+^NK1.1^+^CD49b^+^NKp46^+^ as a mNK population.^42^ Expression of CD43 and CD11b (also called Itgam or Mac-1) defines terminally mature NK cell maturation.^43^ Depending on the expression of CD11b and CD27, NK cell maturation from iNK to mNK can also be divided into four continuous stages: from CD11b^−^CD27^−^ (DN) to CD11b^−^CD27^+^ to CD11b^+^CD27^+^ (DP) and to CD11b^+^CD27^−/low^.^44,45^ In mNK cells, acquisition of inhibitory Ly49 receptors, including Ly49A, Ly49C/I, Ly49G, and NKG2A, can define functional license (Fig. 2a).^46^

Human NK cells may be able to develop not only in the BM but also in extramedullary tissues, such as tonsils and lymph nodes.^47^ The developmental tree of human NK cells is still challenging, especially in relation to ILC subsets, however.^6,48^ The prevailing linear model posits that human NK cells develop along a continuum in which CLPs gradually downregulate CD34 and upregulate CD56.^6,48^ HSCs (Lin^−^CD34^+^CD38^low/−^CD45RA^−^CD10^−^) differentiate into multipotential progenitors (MPP, Lin^−^CD34^+^CD38^−^CD90^−^CD45RA^+^) and then transition into CLPs (Lin^−^CD34^low^CD38^+^CD45RA^+^CD10^+^) with the potential to commit to Pro-B, Pre-T, NKPs (Lin^−^IL-1R1^+^CD122^+^CD38^+^CD123^−^CD45RA^+^CD7^+^) and other ILC progenitors.^6,48^ Acquisition of IL-1R1 marks the earlier stages of committed NKPs, while the appearance of CD122 indicates irreversible lineage specification of NK cells from CLPs. NKPs differentiate into iNK cells, characterized by higher expression of IL-1R1 and the appearance of CD314 (NKG2D), CD335 (NKp46), CD337 (NKp30), and CD161 (the human homolog of the mouse NK1.1). The next transitional stage is the appearance of CD56^bright^ NK cells, marked by high expression of CD56 along with maximal expression of NKG2D, NKp46, NKp30, and CD161. Lastly, CD56^bright^ NK cells transition to CD56^dim^ NK cells, marked by decreased CD56 expression and increased CD16 expression along with the expression of distinct subtypes of CD158 (KIR).^6,48^ According to expression levels of CD56 and CD16, mature NK cells from peripheral blood of healthy individuals can be divided mainly into two subgroups: CD56^bright^CD16^dim^ and CD56^dim^CD16^bright^. The former strongly secrete cytokines upon cytokine stimulation, while the latter have greater cytotoxicity in the resting state.^49–51^ CD56^bright^ NK cells reside primarily in secondary lymphoid tissues, representing 5% of total NK cells, while CD56^dim^ NK cells represent 95% of total NK cells in the circulation.^50–53^ However, a non-linear model of human NK cell development has been proposed in which common myeloid progenitors (CMPs) and granulocyte-monocyte progenitors (GMPs)—isolated from cord blood or human iPSC-derived HOXA^+^CD34^+^ progenitors—can efficiently differentiate into NK cells when cultured in the presence of NK-supporting cytokines and stroma cells.^48,54,55^ Also, some studies support the idea that CD56 ^bright^ and CD56^dim^ NK cells have different ontogenies and that NKPs can directly generate CD56 ^bright^ and CD56^dim^ NK cells (Fig. 2b).^48,56,57^

The activation of NK cells and their cytotoxic attack of target cells is immediate, as it does not require prior antigen presentation or major histocompatibility complex (MHC)-restriction. When NK cells encounter tumor cells, their activation depends on the balance between activating and inhibitory signals that are produced as the various NK cell receptors interact with their ligands from on target cells.^46,58^ The main activating receptors include natural cytotoxicity triggering receptors (NCRs; NKp30, NKp44, NKp46), killer cell immunoglobulin-like receptors (KIRs: KIR-2DS and KIR-3DS), and C-type lectin receptors (NKG2D, CD94/NKG2C, NKG2E/H, and NKG2F). Inhibitory receptors include mainly KIRs (KIR-2DL and KIR-3DL) and C-type lectin receptors (CD94/NKG2A/B).^46,58^ MHC class I (MHC-I) molecules, which are present on most host healthy cells, can bind to KIR-2DL, KIR-3DL or CD94/NKG2A/B on NK cells to avoid NK cell-mediated killing. In mice, NK cells use the Ly49 family of lectin-like receptors instead of the KIRs used by humans.^46,58^

NK cells become activated when they lose their inhibitory signals upon encountering target cells that downregulate MHC-I expression. Activated NK cells eliminate target cells mainly by directly releasing perforin and granzymes, which lyse tumor cells. NK cells can also exert antibody-dependent cellular cytotoxicity (ADCC) via the membrane receptor CD16, or use Fas ligand (FasL) or TNF-related apoptosis-inducing ligand (TRAIL) to induce target cell apoptotic pathways.^29^

## NK cell trafficking and homing at steady state

NK cells develop from HSCs in a continuous process and in a specialized niche, the BM parenchyma, which is localized in perivascular regions proximal to the sinusoidal vessels in the BM. Upon differentiation, immature and some mature NK cells migrate from the parenchyma to the sinusoids and eventually into the bloodstream. Then they traffic into secondary lymphoid tissues and various other tissues at different stages of development, differentiation, and activation. Peculiar subsets of NK cells home back into the specialized niche in the BM to perform specific functions, such as surveillance and control of malignant cells.^59,60^

### Overview of molecules responsible for NK cell homing and trafficking

**Fig. 3:**
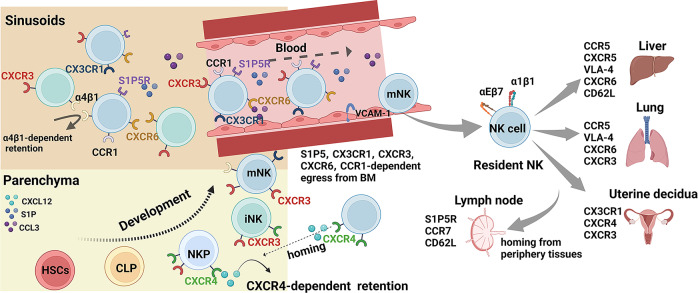
NK cell trafficking and homing at steady state. NK cells develop from HSCs in the BM parenchyma and gradually reach blood and peripheral tissues as they mature. CXCR4 is highly expressed in immature NK cells, including NKPs and iNKs, making NK cell remains in or homes back to the BM parenchyma. When CXCR4 expression is downregulated and the expression of CXCR3, CXCR6, S1P5 and CX3CR1 is upregulated, NK cells gradually egress from the BM and enter peripheral tissues via circulating blood. Created with BioRender.com

Factors that control NK cell trafficking and homing include integrins, selectins, and chemokine receptors as well as their corresponding receptors or ligand, signals induced by cytokines, sphingosine-1-phosphate (S1P), and cellular microenvironments (summarized in Fig. 3). Integrins and chemokine receptors are commonly used as biomarkers for describing circulating and tissue-resident subsets.^61^

Integrins are a group of transmembrane receptors, including 18 reported α subunits and 8 reported β subunits. Each α subunit can bind with multiple β subunits to form various integrin heterodimers.^61^ To communicate with the extracellular matrix (ECM) and other cells, each integrin heterodimer can bind to multiple extracellular binding partner, including over 20 different ECM components, selectins, and cell adhesion molecules (CAMs).^61^ The structures and functions of integrins are highly modular and adaptable, enabling cells to change their size and therefore their trafficking speed.^61^ A variety of integrins expressed on NK cells direct the cells’ migration and tissue residency^61^ and are usually used as markers for distinguishing tissue resident or non-resident subsets of NK cells. β2 integrins are lymphocyte specific, and they enable NK cells to migrate in and out of the circulation and also through tissues. β2 integrins also mediate adhesion and immune synapse formation during the lysis of target cells.^62^ For example, αL (LFA-1)/β2, αM (Mac-1)/β2, αX (CR4)/β2, and αDβ2 expressed on NK cells in blood and lymph nodes can bind CAM family proteins (including vascular cell adhesion molecule-1 (VCAM), ICAM, MadCAM, etc.) in ECM or on non-lymphocytes. As a result, NK cell recruitment from the circulation to enter underlying tissues.^61^ β1 integrins are highly expressed on—but are not specific to—leukocytes. They mediate both leukocytes navigation to tissue microenvironments and also mediate target cell cytotoxicity. For example, αL (LFA-1)/β2, αM (Mac-1)/β2, α1 (VLA-1)/β1, α2 (VLA-2)/β1, α4 (VLA-4)/β1, and α5 (VLA-5)/β1 are critical signatures of tissue-resident cells, and are commonly expressed on NK cells. They can bind collagen, laminin, E-cadherin, fibronectin, VCAM-1, MAdCAM-1, and vitronectin.^61,63,64^ In addition, the binding of α1/β1 to its partner collagen IV or laminin, or the binding of αE(CD103)/β7 to its partner E-cadherin, or the binding of α4/β7 to its partner MadCAM-1, VCAM-1 and fibronectin is responsible for the tissue residency of NK cells, including in liver, lung, tonsil, uterus, skin, kidney, bone, and spleen. This illustrates that integrins adapt to specific tissue microenvironments and to the development and functions of NK cells.^61^

Chemokine receptors (G-protein-coupled seven-transmembrane receptors), in concert with selectin, are key regulators of the integrin activation and switch modes of cell migration. After binding to chemokines, chemokine receptors rapidly modulate integrins’ affinity and clustering as well as actin remodeling, thus directing cell migration.^61^ The four types of chemokine receptors are grouped by structure: C-X-C chemokine receptor (CXCR), C-C chemokine receptor (CCR), C-X3-C chemokine receptor (CX3CR), and XCR. Each has its corresponding chemokine ligands.^61^ Several chemokine receptors play key roles in NK cell trafficking and function, including CXCR1, CXCR3, CXCR4, CXCR6, CCR7, C–C motif chemokine ligand (CCL) 3/4 (also called as macrophage inflammatory protein-1 alpha/beta, MIP-1a/b), CCL5 (also called as regulated activation, normal T-cell expressed, and secreted, RANTES), and C-X-C motif chemokine ligand (CXCL) 1 (also called as activation induced, T-cell derived, and chemokine-related cytokine, ATAC).^61^ Other molecules, such as CD69 and sphingosine-1-pentaphosphate 5 receptor (S1P5), are also reported to regulate NK cell homing and trafficking.^5,59^ In the following section, we consider how the above molecules direct NK cell homing and trafficking in various tissues or organs.

### NK cell trafficking and homing within the BM

NK cells can be found in both the parenchyma and sinusoids of the BM. As they egress, they move first from the parenchyma to the sinusoids and then from the sinusoids into blood. They can remain in the parenchyma only if they express CXCR4, a specific receptor for CXCL12. CXCR4 is highly expressed in immature NK cells, including NKPs and iNKs, and it decreases as NK cells mature.^65,66^ When CXCR4 is antagonized in mice, NK cells are rapidly mobilized from the parenchyma to the sinusoids, and a large number egress from the BM to the periphery.^65^ This suggests that, during maturation of NK cells, decreased CXCR4 expression promotes egress from the BM. In NOD/SCID mice with a reconstructed human immune system, CXCR4 also helps retain human NK cells in the BM.^67,68^ Increased expression of CXCR4 in human CAR NK cells enhances their homing to the BM in NOD-SCID IL2Rγnull (NSG) mice after adoptive transfer.^69,70^

In mice, CX3CR1 is expressed prevalently on terminally mature killer cell lectin-like receptor G1 (KLRG1)^+^ NK cells.^71^ KLRG1^+^CX3CR1^+^ NK cells originate from KLRG1^+^CX3CR1^−^ NK cells.^71^ In the BM, KLRG1^+^CX3CR1^−^ NK cells show higher CXCR4 expression and are localized mainly in the parenchyma.^71^ In contrast, KLRG1^+^CX3CR1^+^ NK cells show reduced CXCR4 expression and are positioned mainly in the sinusoids.^71^ In the absence of CX3CR1, KLRG1^+^ NK cells accumulate in the parenchyma at steady state.^72^ In humans, single-cell RNA sequencing reveals that functionally mature NK cells highly express CX3CR1 and HAVCR2 (TIM-3).^73,74^ These results suggest that CX3CR1 regulates the withdrawal of NK cells from the BM and guides them into the circulation at steady state.

The interaction between VCAM-1 and VLA-4, a heterodimer of integrin α4 (VLA-4/CD49d) and β1 (CD29), is important for maintaining HSCs and B cells in the BM sinusoids^75,76^ and also for retaining NK cells in the BM.^71^ In mice, neutralizing integrin α4 with monoclonal antibodies selectively reduces NK cell numbers in the BM’s sinusoid cavity. Integrin α4 blockage also dramatically reduces the number of KLRG1^+^CX3CR1^+^ NK cells inside the sinusoidal compartment.^71^ One-year treatment of multiple sclerosis patients with the humanized monoclonal antibody natalizumab, which targets the α4 chain of α4β1 and α4β7 integrins, almost doubled the number of NK cells in peripheral blood, and the number decreased when treatment was withdrawn.^77,78^ These results show that VLA-4 is needed to retain NK cells in the sinusoids.

In mice, CXCR3 is more highly expressed in the CD11b^+^CD27^+^ NK cell subset than in other NK cell subsets, and it drives NK cell-specific chemotaxis toward the CXCR3 ligands CXCL10 (IP-10) and CXCL11 (I-TAC).^45^ At steady state in mice, CXCR3 deficiency results in a dearth of NK cells in peripheral tissues, including lung, liver, and peripheral blood, suggesting that CXCR3 affects NK cells in the BM.^79^ In a recent study with a multiple myeloma mouse model involving adoptive transfer, CXCR3^−^ NK cells showed more homing to the BM than CXCR3^+^ NK cells, whereas blocking CXCR3 promoted homing to the BM.^80^ However, CXCR3-deficient NK cells could still egress from the BM—but not from the spleen—into peripheral blood if they were stimulated with IFN-γ or IL-18.^81^ Owing to the lack of direct evidence that NK cells accumulate in the BM parenchyma or sinusoids in CXCR3^−/−^ mice, the role of CXCR3 in the trafficking and homing of NK cells into the BM requires further exploration.

### NK cell egress from the BM

After passing through the BM sinusoids, NK cells enter the blood circulation to migrate to secondary lymphoid organs and peripheral tissues. Several studies have demonstrated that S1P5 and CX3CR1 play important roles in this egression.^71,72,82,83^ During NK cell maturation, expression of S1P5 and CX3CR1 is upregulated in parallel with a progressive decrease in CXCR4 expression.^65,84^

S1P is a lysophospholipid that regulates many biological functions within and outside cells. S1P levels are relatively high in blood and LNs and low in other lymphoid tissues. This concentration gradient between the peripheral circulation and tissues allows cells expressing S1P receptors to flow out of those tissues.^85^ Among the five S1P receptors, S1P5 is specifically expressed in both human and mouse NK cells.^82^ Although NK cells are not completely absent from all organs of S1P5-deficient mice, their distribution is significantly different from that in wild-type mice, as their percentages are significantly lower in the spleen, lung, and peripheral blood. In contrast, the percentages of NK cells in the BM and LNs of S1P5-deficient mice are twice as high as in wild-type mice.^82,83^ Subsequent studies have demonstrated that the role of S1P5 is specific to the egress of NK cells—but not T and B cells—from the BM and LNs. It is also more important for the egress of mature NK cells.^65^ This migration is driven by a concentration gradient of S1P5, both from the BM and LNs.^83^ Although S1P1 may be expressed at low levels on NK cells, inhibiting it with FTY720 in vivo produces a mild accumulation of NK cells in the BM but does not block NK cell egress from the BM or LNs.^83^ In humans, newly exposed to fingolimod, which blocks the S1P receptor, 40% to 50% fewer CD56^bright^ NK cells are detected in peripheral blood 6 h after fingolimod is administered.^86^

CXCR6 was first thought to cause retention of NK cells in the liver, and recent single-cell RNA sequencing revealed that CXCR6^+^ NK cell subsets are preferentially located in liver and are essential for the persistence of memory NK cells.^87,88^ According to another recent study, however, ILC progenitor cells require CXCR6 to leave the BM.^89^ CXCR6 is expressed mainly on NK progenitors and immature NK cells, and CXCR6^−/−^ mice have more of those cells in the BM at steady state.^90^ Also, CXCR6-deficient progenitors are less able to reconstitute the peripheral compartment of NK cells compared with their wild-type counterparts.^90^ This suggests that NK progenitors and immature NK cells need CXCR6 to exit the BM.

### NK cell migration into various normal peripheral tissues

Egress of NK cells into blood from the BM and their eventual entry into peripheral tissues to further mature and create tissue heterogeneity involves the homing of NK cells. Tissue-resident CD56^bright^ NK cells dominate in the gut, tonsils, lymph nodes, and skin, whereas CD56^dim^ NK cells are more frequent in the lungs and liver. In some tissues, tissue-resident NK cells show surface expression of CD69, CD103, and CD49a.^5^ Molecules such as integrins and chemokine receptors and their corresponding receptors or chemokines, which are commonly responsible for NK cell recruitment into all the described peripheral tissues were discussed in the above section: *Overview of molecules responsible for NK cell homing and trafficking*.^61^ In this section, we focus on tissue-specific molecules responsible for NK cell recruitment into peripheral tissues or organs, including liver, lung, tonsil, decidua, and intestine.

In the liver, NK cells account for 30% to 40% of all lymphocytes. Liver ECM is rich in type IV collagen,^91^ which shows high binding affinity with α1/β1 integrins that are highly expressed on mouse liver-resident NK cells.^61,91–93^ Liver NK cells can also be derived from circulating NK cells. VLA-4 mediates the recruitment of NK cells to the liver by binding to VCAM-1, which is expressed on tissue-resident cells such as endothelial cells.^94^ CXCR6 is involved in the trafficking of NK and T cells to the liver, and 35% to 55% of liver NK cells are CXCR6^+^, while only 3% to 5% of spleen NK cells are positive.^88,95,96^ CXCR6 can bind CXCL16 in hepatic sinusoids,^95^ which may promote the retention of peripheral NK cells. During viral infection, NK cells enter the liver in a CXCR6-dependent manner and remain inthere.^87^ In addition, CXCR6 can activate integrin α4 (VLA-4)/β1, which also helps recruit peripheral NK cells to the liver by binding liver VCAM-1. Once there, the former peripheral NK cells can further upregulate αEβ7 and α1/β1 on NK cells that reside in the liver.^97,98^ CCR5, which is expressed on immune cells, has been shown to direct lymphocytes toward inflammatory sites.^99^ Knocking out CCR5 from expanded NK cells (using CRISPR/Cas9 technology) reduces trafficking into liver tissue but increases the number of NK cells in the circulation and lung following adoptive transfer into immunodeficient mice.^100^ Human Eomes^hi^ NK cells show high liver residency, whereas circulating Eomes^low^ NK cells can upregulate Eomes expression if stimulated by cytokines such as IL-15 and transforming growth factor-β (TGF-β) released during inflammation. Upregulated Eomes is believed to increase the expression of CCR5 on NK cells.^97^ However, with increased understanding of liver-resident NK cells and the emergence of the nomenclature ILC1, the previous characterization of liver bulk NK cells needs to be updated with a description that is distinct from ILC1s.^101^

In humans, the major population of lung NK cells is CD56^bright^CD16^−^ and they show a relatively poor response to target cells but produce more cytokines than peripheral blood NK cells.^102^ Tissue-resident NK cells in the lung also highly express CCL5, MIP-1β, and granulocyte-macrophage colony-stimulating factor (GM-CSF), which are chemokines that shape cell migration and recruit other immune cells.^103^ In the lung, tissue-resident NK cells expressing αEβ7 are located in the alveolar epithelium, where they bind to E-cadherin. In contrast, α1β1^high^ NK cells are preferentially located in the basement membrane, which is rich in type IV collagen.^61^ VLA-4 mediates the recruitment of NK cells to the lung by binding to VCAM-1, which is expressed on tissue-resident or target cells such as endothelial cells and tumor cells.^94^ CCR5 and its ligands CCL3, CCL4, and CCL5, are rapidly upregulated during influenza infection, contributing to extensive recruitment of NK cells, as well as other inflammatory cells to the lung.^104^ Single-cell RNA-sequencing data from patients with severe acute respiratory syndrome coronavirus 2 (SARS-CoV-2) infection indicate that the transcription of CXCR3, CXCR6, and CCR5 is greater in NK cells of Coronavirus Disease 2019 (COVID-19) patients with moderate disease than in those with severe disease.^105^

In addition to other secondary lymphoid tissues, the tonsil is also an important site for the development and residence of human NK cells,^106–108^ though their etiology has been debated in the last decade. One early NK cell precursor expressing integrin β7 is considered to seed in tonsil after exiting the BM, as it is also found in peripheral blood expressing L-selectin, which help recruit this precursor to tissues.^106,109^ Tonsil NK cells express high levels of αE, β7, and α1 integrins along with lower levels of αM and αX; these cells are named α1^hi^αE^hi^αX^lo^αM^lo^ NK cells. In addition to retaining NK cells in the liver, CXCR6 also helps confer specificity to epithelial tissue.^87,110,111^ Tonsil α1^hi^αE^hi^αX^lo^αM^lo^ NK cells are close to tonsillar epithelium and also express high levels of CXCR6,^112^ indicating that CXCR6 might help recruit NK cells to tonsils.

NK cells are the most abundant lymphocytes in the decidua, representing 50% to 90% of total decidual lymphoid cells, which have pivotal roles throughout pregnancy. Unlike peripheral blood NK cells, most decidual NK (dNK) cells are CD56^bright^ and α1 integrin positive, and they are less cytotoxic but produce large amounts of IFN-γ, vascular endothelial growth factor (VEGF), and IL-8.^113^ dNK cells induce trophoblast invasion, decidual transformation, vascularization, and placental formation and also fight against placental infection by expressing cytokines or chemokines or recruiting other cells.^113^ Aberrant dNK cell activation induces breakdown of tolerance of the maternal–fetal interface, which can lead to preeclampsia, recurrent spontaneous abortion, endometriosis, recurrent implantation failure, and preterm birth.^113^ Although the origin of dNK cells is still under debate—recruitment from peripheral NK cells *vs*. differentiation in situ from progenitor cells—some evidence supports the idea that NK cells from peripheral blood are recruited into the uterus.^113^ dNK cells show high expression levels of CXCR3, a relatively lower level of CXCR4, and very low levels of CXCR1, CXCR2, CX3CR1, or CCR1, 2, 3. Endometrial or trophoblast cells express chemokines, including CXCL10, CXCL12, CCL3, and CX3CL1, which induce the recruitment of NK cells from peripheral blood toward decidua mainly via interaction with CXCR3 and CXCR4.^114,115^ Chemerin induces NK cell migration toward decidual stromal cells via the chemerin receptor (ChemR23).^116^ Estradiol and progesterone treatments promote the expression of CXCL10 and CXCL11 (ligands for CXCR3) in endometrial samples in an in vitro organ culture system.^117^ In a mouse model of IFN-γ-induced abortion, IFN-γ significantly increases the expression of CX3CL1 in the uterus, which recruits CD49b^+^ NK cells via CX3CR1 to the uterus and eventually provokes fetal loss.^118^

In the intestine, NK cells locate in both epithelium and lamina propria. In epithelium, tissue-resident NK cell expressing high αEβ7 bind to E-cadherin at cell junctions, whereas NK cells upregulate α1/β1 binding to fibronectin, collagen, and laminin in the lamina propria.^61,119,120^ αE (also called as CD103) is a marker of intraepithelial localization in the gut. TGF-β induces the expression of CD103 in ILCs, indicating that gut NK cells are imprinted by TGF-β, which could explain their reduced expression of cytotoxic molecules compared with peripheral blood NK cells.^112,121^ A recent integrating high-dimensional analysis of NK cells reveals that human intestinal NK cells contains of ~40% of the CD56^bright^CD16^−^ subset. This subset does not express the CD16 high-affinity IgG receptor, but highly expresses CD69 and CD103.^122^ Expression of thymus-expressed chemokine (TECK, also called as CCL25) is highly restricted to the epithelium of the small intestine, where it mediates the recruitment of CD4^+^ and CD8^+^ T cells via its ligand CCR9.^123–125^ A previous study revealed that peripheral blood NK cells showed surface expression of CCR9 in healthy donors,^126^ suggesting that CCL25-CCR9 might possibly regulate the chemotaxis of NK cells in the small intestine.

### NK cell homing and egress in secondary lymphoid tissues (SLT)

In addition to extravasation from peripheral blood to various solid tissues, NK cells may eventually egress from peripheral tissues and home to SLT. NK cells traffic to LNs through high endothelial venules in the medulla and paracortical areas, where they interact with DCs. CD62L mediates the initial interaction between leukocytes and vascular endothelium, the first step in the extravasation of leukocytes into tissues. In mice, CD62L is required for NK cell homing and re-recruitment of both resting and activated regional LNs; it binds to CD62L ligand expressed on endothelial cells.^127,128^ CD62L-mediated NK cell recruitment to activated regional LNs is critical for restricting tumor metastasis into secondary lymphoid organs.^128^ One recent study revealed that CD62L is also expressed on precursors to all ILC subsets in both humans and mice and that it is required for their entry into LNs.^129^ In humans, CD56^bright^ NK cells exhibit relatively higher expression of CD62L than do CD56^dim^ NK cells, T cells, B cells, neutrophils, and monocytes.^129^ An ex vivo study found that human CD56^bright^ NK cells show highly efficient adhesion to HEV in BALB/c lymph node tissue sections in a CD62L-dependent manner.^130^

CCR7 is a well-known chemotactic receptor that directs adaptive and innate immune cells to secondary lymphoid tissue.^131,132^ At steady state, CCR7 is expressed almost on all human CD56^bright^ NK cells but not on CD56^dim^ NK cells, and it induces the homing of human NK cells to LNs.^126^ In vitro expansion of NK cells with K562 feeder cells expressing CCR7 reveals transfer of CCR7 from the feeder cells onto the surface of NK cells via trogocytosis, as we recently showed for TYRO3.^133^ Lymph node homing of NK cells that had acquired CCR7 via trogocytosis increased by 144% in athymic nude mice.^134^ This demonstrates that CCR7 is also important for the homing of human NK cells to lymph nodes. A recent study also showed that all ILCs, especially ILC1s, are recruited to LNs in a CCR7- and CD62L-dependent manner.^132^ However, there is still no direct evidence for how CCR7 contributes to the homing and trafficking of murine NK cells.

NK cells in SLT and efferent lymph fluid display slightly different phenotypes.^51^ However, there is very little information regarding the mechanisms underlying NK cells’ egression from SLTs, though S1P5 is shown to be more important for egression from the SLT than from the BM.^65^ Expression of SPNS2 (an S1P transporter required in lymphatic endothelial cells) and S1P5 is required to localize NK cells at the medullary cords of LNs, and deficiency of either causes NK cells to appear in the T-cell zone of LNs.^65,135,136^

## Trafficking of NK cells into tumors

### General overview of NK cells in the TME

**Fig. 4:**
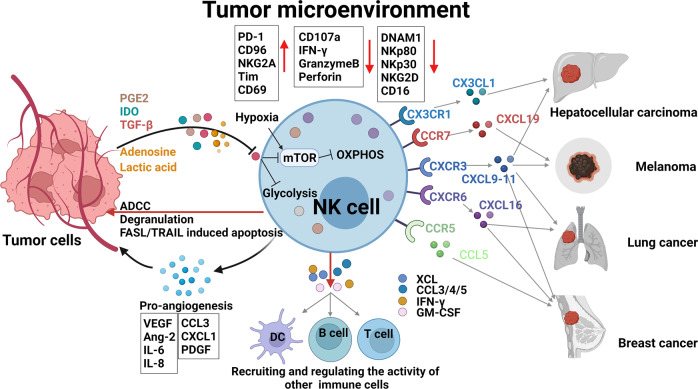
NK cells in the TME. NK cells extravasate from the blood, traverse the ECM and tumor stroma, and reach the tumor bed when they are recruited by integrin, chemokine receptor, and selectin. In the TME, NK cells eliminate tumor via degranulation, ADCC, or FASL/TRAIL-induced apoptosis. NK cells can also secrete cytokines or chemokines to recruit other immune cells and upregulate the anti-tumor response. However, NK cell responsiveness is often hindered by repressive factors secreted by tumor cells or other cells or by direct cell–cell interaction. In addition, NK cells can be educated by tumor cells, for example, to secrete pro-angiogenic factors to promote tumor angiogenesis. This education can switch anti-tumor immunity to pro-tumor immunity in the TME. Created with BioRender.com

Several recent reviews have detailed interactions between NK cells and tumors.^13,14,31,32,137^ NK cells are commonly found in the TME of human tumors, including primary tumors, metastases, and tumor-infiltrated lymph nodes. NK cells can easily reach hematopoietic tumors in peripheral blood. However, it is challenging to reach and infiltrate solid tumors.^31,32,137^ The density of endogenous NK cells is extremely low, with no >100 cells per mm^2^, in contrast to several hundred CD8^+^ T cells per mm^2^ in various human solid tumors.^33,138^ CD56 ^bright^ NK cells, which are less cytotoxic, are more enriched in various tumors than in most normal peripheral tissues.^53^ Nonetheless, clinical statistics suggest that the abundance of NK cells in the TME predicts better outcomes in patients with several types of cancer, including hepatocellular carcinoma (HCC),^139^ melanoma,^16,140,141^ breast cancer,^18^ non-small cell lung cancer (NSCLC),^142^ squamous cell lung cancer,^143^ pulmonary adenocarcinoma,^144^ RCC,^145^ and gastric cancer.^146^ Such infiltration not only enhances direct killing of target cells but also provides immunomodulatory cytokines, which in turn shape adaptive immune responses.^140,147–149^ To reach a solid tumor bed, NK cells must first extravasate from the blood, and then traverse the ECM and tumor stromal by degrading ECM with matrix metalloproteinases, urokinase plasminogen activator and serine dipeptidyl peptidase IV.^150^ Several chemokine–chemokine receptor axes have been reported to be responsible for recruiting both human and murine NK cells to the TME, including CCL5-CCR5, CCL27-CCR10, and CX3CL1-CX3CR1. We discuss these in detail for various types of cancer in the following sections (Summarized in Fig. 4).

After NK cells reach the tumor bed, integration of complex signals from multiple ligand–receptor interactions lead to NK cell recognition and activation, either after cell–cell contact or when the cells act independently.^31,32,137^ The loss or aberrant expression of MHC-I molecules on tumor cells are the most important signal for NK cell recognition.^31,32,137^ Activated NK cells can lyse tumor cells by directly releasing perforin and granzymes and inducing apoptosis with ADCC, FasL, or TRAIL.^13,29^ When NK cells kill tumor cells, tumor antigens that can prime adaptive immune responses are released.^13,29^ One piece of direct evidence that NK cells regulate adaptive immune responses is that adoptive transfer of iPSC-NK cells can recruit T cells and cooperate with them to respond to anti-PD-1 antibodies in solid tumor.^151^ Activated NK cells can also secrete various cytokines, including IFN-γ, GM-CSF, G-CSF, M-CSF, TNF, IL-5, IL-10, IL-13, FLT3LG, TGF-α, XCL, CCL3/4/5, and so on, which recruit other immune cells and regulate their anti-tumor responses.^152^ The ability of IFN-γ to shape the adaptive immune response is the best studied. This cytokine acts on a lot of immune cells, including macrophages, DCs, T cells, B cells, and even NK cells themselves [detailed in ref. ^153^].^153^ NK cells can also directly kill cancer stem cells or undifferentiated tumors and trigger the differentiation of cancer stem cells or undifferentiated tumors by secreting IFN-γ.^31,154^ NK cells also produce an abundance of chemokines, such as CCL5 and XCL1/2, that recruit cDC1s, an anti-tumor immune subset.^155,156^ Using an AML model, we discovered that ILC1s, which used to be recognized as a subset of NK cells, target leukemia stem cells more potently than NK cells.^157^

NK cell responsiveness is often hindered by the immunosuppressive TME. After they infiltrate into the TME, their phenotype and metabolism alter, impairing their cytotoxicity, decreasing their expression of activating receptors (including DNAM1, NKp80, NKp30, and CD16), and increasing their expression of molecules related to NK cell exhaustion (including PD-1, CD96, Tim3, and TIGIT).^31,158,159^ A series of soluble molecules such as TGF-β, IL-10, indoleamine 2,3-dioxygenase (IDO), and prostaglandin E2 (PGE2) produced by tumor cells, Treg cells, carcinoma-associated fibroblasts (CAFs), or other cells can also suppress NK cells effector functions.^31,32,137^ Moreover, immunosuppressive cells including Tregs and myeloid-derived suppressive cells (MDSC), tumor cells, or other immune cells can impair NK cell effector functions through receptor-ligand-mediated interactions (e.g., NKG2A-HLA-E, 2B4-CD48). In addition, tumor cells can express cell-released forms of KIR or NCR ligand, including platelet-derived growth factor (PDGF)-DD and nidogen-1 (NID1) glycoprotein (for NKp44), NKG2DL (for NKG2D), and NKp30L (for NKp30), which obstruct the direct interaction between NK cells and tumor cells [for details, please refer to ref. ^31^].^31^ CD9 on NK cells acquired from tubo-ovarian high-grade serous carcinoma (HGSC) represses cytotoxicity and anti-tumor cytokine production of NK cells, which might be due to reactivation of ADAM17 cleavage activity that sheds NK ligand or receptors (e.g., cleavage of CD16).^160^

During tumor progression, NK cells can also be educated by tumor cells, which can switch anti-tumor immunity to pro-tumor immunity, creating an immune-tolerant microenvironment that facilitates tumor cell growth or metastasis.^161^ For example, murine NK cells isolated from the spleen of mice bearing keratin-14^+^ breast cancer cells show increased expression of inhibitory receptors such as LAG3, LKRG1, and KLRC1. They also promote the formation of tumor cell colonies and organoid invasion when they are co-cultured with tumor organoid in vitro.^162^ Most tumor-infiltrated NK cells show decidual-like NK cell phenotypes (CD56^bright^CD16^dim/−^).When seeded in the uterus, they secrete angiogenic factors, which support angiogenesis by encouraging NK cells to interact with endothelial cells, recruit monocytes, or polarize macrophages.^163^ These decidual-like tumor-infiltrated NK cells showed augmented expression of pro-angiogenesis factors such as VEGF, Ang-2, IL-6, CCL3, CXCL1, CCR7, CD146, CD9, PDGF, and IL-8.^163–166^ Tumor-derived immune suppressors in the TME, such as TGF-β, soluble HLA-G, PGE2, adenosine, extracellular vesicles, and miRNAs, mainly shift tumor-infiltrated NK cells toward a pro-angiogenic polarization [reviewed in ref. ^161^].^161^ In vitro treatment with TGF-β1 transforms peripheral blood NK cells from healthy donors into dNK-like phenotypes, promoting the production of VEGF and platelet-derived growth factor.^165–168^ Cell-intrinsic factors, such as STAT5 and hypoxia inducible factor-1α (HIF-1α), have also been reported to regulate pro-angiogenic phenotypes of NK cells.^169,170^

Here, we review the effects of chemokine receptors and other factors on the infiltration of NK cells in various tumor models, including those for HCC, lung cancer, breast cancer, RCC, and melanoma, as well as in other less-studied models (Table 1 and Fig. 4).

**Table 1:** Chemokine receptors affect the trafficking of NK cells into tumors

Diseases	Chemokine receptor	References
HCC	CXCL9/10 ↑ -CXCR3, CX3CL1 ↑ -CX3CR1, CCL5 ↑ -CCR5, CXCR6?	^174,175,177^
Melanoma	CXCL10-CXCR3 ↑ , CCL19 ↑ -CCR7, CMKLR1 ↑	^182–185,189,193^
Breast cancer	CXCR3 ↑ , CXCL16 ↑ -CXCR6, CCL5 ↑ , CCL2 ↑ , CXCL12-CXCR4 ↓	^199,201,202,204,206^
Lung cancer	CXCL10-CXCR3 ↑ , CX3CL1 ↑ -CX3CR1, CCL5-CCR5 ↓	^212,213,215,216^
RCC	CXCL9/10/11-CXCR3 ↑ , CXCR2 ↑	^218,219^
Colorectal cancer	CCL27/28-CCR10 ↑ , CXCL16-CXCR6 ↑	^221^
Endometrial carcinoma	CXCL12 ↓ , IP-10 ↓ , CCL27 ↓	^222^
Pancreatic ductal adenocarcinoma	CXCL9/10 ↑ -CXCR3	^223^
Glioblastoma	CCL5 ↑ , CXCL10 ↑	^224^

### Trafficking of NK cells in HCC

HCC is a common result of chronic liver infection or inflammation, and NK cell infiltration into the liver is observed in patients with the disase.^171^ In addition to the common suppressive factors that impair NK cell effector functions, there are also some HCC-specific factors. Although lectin-like transcript 1 (LLT1) is reportedly not expressed in normal liver tissues, it is upregulated in HCC to inhibit the cytotoxicity of NK cells.^172^ In addition to dampening the functional activities of NK cells, the hepatic TME also affects the chemotactic phenotype of NK cells, including by downregulating CCR1, CCR5, and CXCR3.^173^ Overexpression of CCL5 in cancer cells by adenovirus increases the chemotaxis of NK-92 cells to the tumor site and enhances their anti-tumor activity.^174^ This further confirms that NK cells can fight HCC by increasing their infiltration, as shown in other studies. Dipeptidyl peptidase 4 enhances the biological activity of the CXCL10 secreted by HCC cells by inhibiting CD26, thus maintaining the chemotactic activity of the CXCR3-CXCL10 axis.^175^ Maintenance of that axis increases the trafficking rate of NK cells, which is beneficial for inhibiting HCC development. This observation confirms the role of CXCR3^+^ NK cells in HCC.^175^ Enhancer of zeste homolog 2 (EZH2) is a histone H3 lysine 27 methyltransferase, which impedes the migration of NK cells by inhibiting the transcription of CXCL10 in tumor cells.^176^ MiR-561-5p controls the infiltration and functions of CX3CR1^+^ NK cells through CX3CL1-dependent regulation. CX3CL1 stimulates the chemotactic migration and cytotoxicity of CX3CR1^+^ NK cells through the STAT3 signaling pathway to control the occurrence and metastasis of HCC.^177^ It is feasible to regulate the migration of NK cells to intratumoral tissues in HCC by targeting CXCR3-CXCL10 and CX3CL1-CX3CR1 through drug stimulation or genetic engineering.

NK cells stimulated by hapten or various viruses return to the liver in a CXCR6-dependent manner and stay there for several months, which may indicate the importance of CXCR6^+^ NK cells in HCC.^87,88^ Interestingly, in a recent study of diethylnitrosamine-induced liver cancer, CXCR6 deficiency reduced intrahepatic numbers of invariant NKT, CD4^+^, and CD8^+^ T cells. Diethylnitrosamine-treated wild-type mice that had received BM from CXCR6-deficient mice developed more liver tumors and fewer invariant NKTs.^178^ Given that CXCR6 is critical for the homing of both NK cells and T cells to the liver, targeting CXCR6 could be an alternative way to improve the efficiency of HCC immunotherapy.

The degree to which tumor-infiltrating CD49a^+^ NK cells accumulate in the liver strongly affects prognosis for patients with HCC.^179^ A comparison of CD49a^+^ and CD49a^−^ NK cell transcripts showed that CD49a^+^ NK cells highly express CXCR6 and CCR3.^179^ However, previously defined liver CD49a^+^ NK cells are now considered to be liver-resident ILC1s that differentiate from liver-resident Lin^−^Sca-1^+^Mac-1^+^ progenitor cells.^36^ The presumed link between the accumulation of CD49a^+^ NK cells (or ILC1s) and prognosis in HCC needs to be reevaluated.

### Trafficking of NK cells in melanoma

Melanoma, one of the most common cancers, is highly metastatic and has high immunogenicity. Melanoma cells escape being killed by NK cells by inhibiting the immune cells’ functional activities.^180^ At present, using adoptive NK cells to increase the migration and infiltration of NK cells into melanoma seems a promising approach.^181^ Previous studies reveal that the accumulation of NK cells in tumors is dependent on the expression of the CXCR3 ligand, CXCL10, in the tumor tissue.^182,183^ CXCR3-deficient murine NK cells showed reduced migration to melanoma cells, decreasing the animals’ survival rates.^182,183^ These results convincingly demonstrate that CXCR3 plays a predominant role in the anti-tumor effects of NK cells. Expression of the CXCR3 receptor in human NK cells expanded in vitro with a high dose of IL-2 (1000 IU/ml) was 10 times greater than in resting NK cells, and the expanded cells showed increased migration toward melanoma cells in a CXCL10-dependent manner.^184^ This suggests that strategies for improving CXCR3 expression on NK cells that have been expanded ex vivo may become potential weapons against melanoma.

As described above, CCR7 is one driver of NK cell homing to secondary lymphoid tissue under steady state or pathological conditions.^131^ The frequency of CCR7^+^ CD56^bright^ NK cells in peripheral blood and the serum concentration of the CCR7 ligand, CCL19, are significantly higher in patients with phase IV melanoma compared to those with stage III melanoma or healthy people.^185^ Such an increase in serum CCL19 may reduce NK cell migration to melanoma-infiltrated LNs. Melanoma cells also express CCR7 and concurrently express PD-L1 and Gal-9, especially when they are metastatic.^185^ In light of the finding that trogocytosis of tumor cell-derived CCR7 to NK cells results in NK cell trafficking from tumor tissue to LNs,^134^ blockage of the CCR7-CCL19 axis may keep more NK cells in the TME. Thus, inhibiting both the CCR7 and PD-L1 that are expressed on tumor cells can be considered a new approach to treating melanoma.

CCL5 displays chemotactic activity and induces the migration of several immune cell subsets to inflammatory sites because it can bind to three different C-C chemokine receptors—CCR1, CCR3, and CCR5. CCL5 is released by both tumor cells and immune cells, including NK and T cells, and higher levels of CCL5 in melanoma patients correlate with significantly increased survival.^186^ Expression of CCL5 also correlates strongly and positively with that of NK cell markers in human melanoma tumors,^186–188^ and that association is very significant, as verified in other melanoma studies.^189,190^ Melanoma cells deficient in the growth factor progranulin show increased CCL5 expression and enhanced infiltration of NK cells but not T cells. Silencing CCL5 in progranulin-deficient melanoma cells reverses the increased recruitment of NK cells.^189^ Certain viruses are potent tools for tumor-specific immune activation. Infecting human melanoma cells with lymphocytic choriomeningitis virus (LCMV), a non-oncolytic virus, results in fast tumor regression and enhances NK cell infiltration to the tumor site, and the outcome is dependent on CCL5.^190^

ChemR23, a receptor for chemerin (also called retinoic acid receptor responder protein 2, RARRES2), is expressed on NK cells. The chemotactic activity of the chemerin-ChemR23 axis was first reported to mediate the migration of plasmacytoid DCs and NK cells involved in autoimmune diseases.^191,192^ Further study revealed that chemerin was downregulated during melanoma growth and that it suppresses melanoma growth by recruiting NK cells in a ChemR23-dependent manner.^193^ Recent studies revealed that all-trans retinoic acid, a potent inducer of chemerin, enhances the recruitment of ChemR23-dependent NK cells and suppresses melanoma growth.^194^

In addition to the interactions between the chemotactic receptors of NK cells and some chemokines secreted by melanoma cells, other internal and external factors associated with the disease also affect the infiltration of NK cells. IL-32, a pro-inflammatory cytokine, is expressed in various cancers and immune cells. A positive correlation between IL-32 expression and infiltration of active NK cells was found in cutaneous melanoma.^195^ In a mouse model geared to understanding interactions between the immune system and the TME, the formyl peptide receptor (FPR) agonist WKYMVm promoted the migration of NK cells toward B16 melanoma cells and repressed tumor growth, whereas the FPR antagonist WRW (4) had the opposite effect.^196^

### Trafficking of NK cells in breast cancer

Breast cancer is one of the most common cancers in women worldwide, and about 70% to 80% of patients with early stage (nonmetastatic) disease are curable. In contrast, advanced breast cancer with distant organ metastases is still incurable.^197^ NK cells not only inhibit the occurrence and development of breast cancer but also help control the hematological spread of related cancer stem cells.^198^ The number of tumor-infiltrating NK cells correlates positively with overall survival rates of breast cancer patients and correlates negatively with tumor-draining lymph node metastasis.^199,200^

In an analysis of tumor-infiltrating NK cell subsets in patients with breast cancer, CXCR3^+^ NK cells accounted for 60% of total NK cells. The proportion of tumor-infiltrating CXCR3^+^ NK cells was lower in patients with LN metastasis than in those without, indicating the importance of CXCR3^+^ in tumor development.^199^ In the early study, irradiation enhanced the expression of CXCL16 in breast cancer cells and increased the migration of activated NK cells expressing CXCR6 into breast cancer tissue, thus enhancing the NK cells’ anti-tumor function.^201^

Triple-negative breast cancer is the most aggressive form of the disease. Evaluating a retrospective cohort and publicly available data sets of 72 patients with triple-negative breast cancer revealed that high expression of CCL5 related positively to recruitment of NK cells, reflecting the importance of CCL5 in regulating NK cells in breast cancer.^202^ In an animal model of triple-negative breast cancer, NK cells activated by cationic nanoparticles effectively inhibited tumor growth by upregulating the expression of CXCR4 and CCR4.^203^

Under normal physiological conditions, the presence or absence of *miR-155* has no effect on the number of NK cells and cytotoxic receptors in mice. However, the absence of *miR-155* weakens the response of NK cells to CCL2 chemokines in vitro. When *miR-155*^−/−^ mice were attacked by mammary carcinomas, the increased tumor burden was related directly to the decrease in tumor-infiltrating NK cells. It is suggested that *miR-155* may promote chemotaxis by regulating the receptors of CCL2 on NK cells.^204^ In mesenchymal stem cells, Sirt1, the closest mammalian homolog of yeast Sir2, promoted the expression of CXCL10, which then recruited NK cells to effectively inhibit tumor growth.^205^ CXCL12, a chemokine secreted by hepatic stellate cells, induced NK cell quiescence through its receptor, CXCR4, promoting breast cancer outgrowth.^206^ Another recent study revealed that a ruthenium polypyridyl complex increased the production of NKG2D ligands by breast cancer cells, in turn promoting the infiltration of adoptively transferred NK cells and enhancing their therapeutic effect on breast tumors in vivo.^207^

### Trafficking of NK cells in lung cancer

Being open to the environment, lungs often develop tumors and are attacked by microscopic pathogens. So not surprisingly, lung cancer is the leading cause of cancer death among both men and women in the U.S.^208^ NK cells are located mainly in the parenchyma of the lungs, accounting for 10% to 20% of lymphocytes.^209^ In a mouse model of Kras-driven lung cancer, the number of NK cells gradually decreased between tumor initiation and tumor progression.^210^ Also, a transcriptional assay uncovered significant changes in migratory patterns of tumor-infiltrated NK cells in the TME of human NSCLC. These changes included downregulated S1PR1 and CXCR1 and increased expression of CXCR5, CXCR6, and CXCL13 compared with non-tumor NK cells.^211^ In an orthotopic mouse lung tumor model, mature circulating NK cells migrated to tumor-bearing lungs in a CXCR3-dependent manner. However, blockading CXCR3 failed to affect tumor growth, suggesting that tissue-resident NK cells may compensate for decreased migration of NK cells from the circulation.^212^

Some recent studies of the mechanisms behind new strategies for controlling lung cancer have also implicated NK cell migration. Jinfukang, a traditional Chinese medicine, not only increased the cytotoxicity of NK cells but also promoted the expression and secretion of the chemokine CX3CL1 in circulating tumor cells, thus recruiting NK cells and inhibiting lung cancer metastasis.^213^ Systemic delivery of TUSC2 (a tumor suppressor gene) in nanovesicles (as already performed in clinical trials) combined with anti-PD-1 antibody enhanced the proliferation and infiltration of NK cells in a mouse lung cancer model.^214^ In a mouse model of NSCLC, rocaglamide, a natural product, not only inhibited autophagy and restores levels of granzyme B derived from NK cells but also promoted the infiltration of NK cells into tumor tissues. Mechanistically, rocaglamide improves the expression of the chemokines CCL5 and CXCL10 in NSCLC cells.^215,216^ Whether there is a direct relationship between infiltration of NK cells and increased expression of CCL5 and CXCL10 in NSCLC cells remains to be studied.

In summary, the roles and mechanisms relating to NK cell migration in lung cancer are still largely unknown. Nevertheless, it is likely that further in-depth studies could provide opportunities for improving the effectiveness of NK cell-based therapy in lung cancer.

### Trafficking of NK cells in other tumors

RCC is a urinary malignant tumor with high global incidence. NK cells with a migration phenotype are found in RCC tumor tissues compared with healthy kidney tissues.^217^ Increased expression of chemokine-receptor (CXCL9-11/CXCR3) is also detected in RCC.^218^ When NK cells are genetically modified to overexpress CXCR2, their migration and ability to resist RCC cells are enhanced in vitro.^219^

Although NK cells are very scarce in the TME in colorectal cancer, levels of chemokines responsible for NK cell recruitment (including CCL3, CXCL10 and CXCL12) are upregulated.^220^ However, there is limited direct evidence for NK cell recruitment into the TME in colorectal cancer. Human colorectal cancer cells, HCT-116, express CCL27 and CCL28, which mediate chemotaxis by binding to the chemokine receptor CCR10 on NK cells in vitro.^221^ Human colorectal cancer cells strongly express CXCL16, which binds to CXCR6 on NK cells. After NK-92 cells were treated with dimethyl fumaric acid and monomethylfumarate, they showed enhanced expression of CCR10 and CXCR6 and enhanced chemotaxis toward HCT-116 cells in vitro.^221^

An analysis of the TME of endometrial carcinoma found lower than normal levels of chemokines, including CXCL12, CXCL10, and CCL27.^222^ This deficit may potentially reduce the recruitment of NK cells to the tumor site.

Dipeptidyl peptidase inhibitors increased levels of CXCL9/10 on pancreatic cancer cells and recruited CXCR3^+^ NK cells to enhance the immune response to PD-1 blockade.^223^ In glioblastoma, pharmacological inhibition of autophagy by chloroquine promoted the expression of the chemokines CCL5 and CXCL10 by tumor cells, enhancing infiltration of CAR NK cells into the tumor tissues.^224^ We reported that herpes simplex virus 1-based oncolytic virus increased NK cell infiltration in various mouse models of glioblastoma.^225–231^ However, the detailed mechanisms underlying the interaction between chemokines and their receptors have yet to be explored.

## Factors influencing NK cell trafficking and homing

**Fig. 5:**
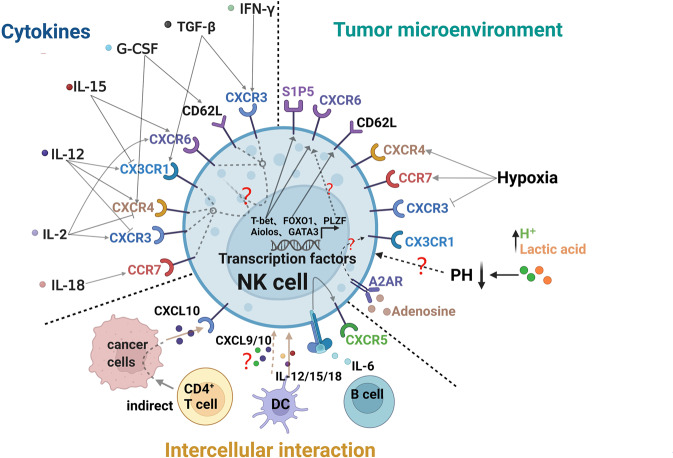
Factors affecting NK cell trafficking and homing. Cell intrinsic factors (e.g., transcription factors), extrinsic factors (e.g., cytokines, hypoxia, low pH), and cell–cell interactions affect NK cell trafficking and homing in both healthy and tumor tissues. During NK cell development, some transcription factors cell-intrinsically regulate the expression of chemokine receptors, selectins, and integrins, etc., thus helping to regulate NK cell homing and trafficking in a cell-intrinsic manner. Cytokines and immune suppressive factors from the TME (e.g., hypoxia, low pH, and adenosine) can also sabotage the recruitment and homing of NK cells in specific tissues by affecting the expression of chemokine receptors, selectins, and integrins on NK cells or their corresponding ligands in the TME. DCs, B cells, and CD4^+^ cells also affect the migration of NK cells via the chemokine–chemokine receptor axis. Created with BioRender.com

Adoptive infusion of NK cells is currently an attractive way to treat cancer, and expanding NK cells ex vivo with cytokines for several weeks before infusion is the predominant way to obtain sufficient cells for clinical use. Therefore, understanding the mechanisms that affect the expression of migration-related receptors and their corresponding ligands should help improve the infiltration of adoptively transferred NK cells into tumor tissue. Here, we summarize some possible factors affecting NK migration, including cell-intrinsic factors (e.g., transcriptional factors), cell-extrinsic factors (e.g., cytokines, hypoxia, low pH), and intercellular interactions (summarized in Fig. 5).

### Cell-intrinsic factors governing NK cell homing and trafficking

During their differentiation and development, NK cells are regulated by various transcription factors, such as T-bet, NFIL3, RUNX3, ID2, FOXO1,^232^ Smad4,^233^ and XBP1s,^234^ etc.^34^ Some transcription factors directly or indirectly promote or inhibit the expression of chemokine receptors, selectins, integrins etc, thus helping to regulate NK cell homing and trafficking in a cell-intrinsic manner (Fig. 5). Expression of some transcriptional factors might also be changed in the TME. We discuss some of them in detail below.

Deficiency of T-bet, a critical transcription factor in NK cells, decreased the number of NK cells in blood and spleen but increased their number in the BM and LNs.^235^ A comparison of the expression of CXCR6, S1P5, and CCR5 between T-bet-deficient and wild-type mice indicated that T-bet promotes the expression of CXCR6 and S1P5, which NK cells need to egress from the BM.^83^ Several repressive factors in the TME, including TGF-β,^236^ inhibit T-bet expression, and impaired T-bet expression in NK cells may promote tumor development and progression by downregulating NK cell recruitment, especially in liver cancer.^237^

The Ikaros family member IKZF3 (Aiolos) is required for terminal maturation of NK cells. Although IKZF3 deletion impaired NK cell terminal maturation and did not affect the cytotoxic function of freshly isolated NK cells in vitro, it enhanced in vivo anti-tumor activity in a mouse lung melanoma metastasis model and tended to increase the ratio of NK cells to tumor cells in the TME.^238^ RNA-seq analysis showed that IKZF3-deficient mice had significantly higher mRNA levels of CXCR2 than wild-type NK cells. Collectively, this evidence indicates that IKZF3 negatively regulates NK cell trafficking.^238^

GATA3 is dispensable for NK cell development but is required for IFN-γ production and controlling infection.^239^ GATA3 deficiency causes NK cells to stay in the BM and specifically reduces NK cells in the liver but not the spleen, suggesting impaired trafficking from BM to the liver.^239^ Accordingly, GATA3-deficient NK cells showed increased CD62L expression but reduced CD11c and VLA-4 expression.^239^ VLA-4 enhanced the recruitment of cells to the liver by binding VCAM-1 onto endothelial cells.^94^ However, the mechanistic details of how GATA3 specifically regulates NK cell trafficking to the liver during tumor development are largely unknown.

We previously identified FOXO1 as a negative transcription factor for NK cell development and effector function. NK cell FOXO1 activity was absent from the TME in a mouse model of lung melanoma metastasis. FOXO1 promoted NK cell homing to peripheral LNs, which might depend on its ability to regulate the expression of CD62L.^232^ Whether FOXO1’s ability to regulate NK cell homing helps promote NK cell infiltration into the TME is still unknown.

One recent study suggested that the transcription factor promyelocytic leukemia zinc finger protein (PLZF) may also regulate the homing of NK cells to the liver, as its expression was higher in intrahepatic CD56^bright^ NK cells—and especially in CXCR6^+^CD69^+^ liver-resident NK cells—than in peripheral blood CD56^bright^ NK cells.^240^ A small population of PLZF^hi^CD56^bright^ NK cells in peripheral blood that also express CXCR6 and CD69 might have been an intermediate population that contributed to the renewal of liver-resident NK cells.^240^ One recent study found that IFN-β treatment reduced the expression of PLZF on peripheral blood NK cells in healthy donors,^152^ suggesting that PLZF might help recruit NK cells to the liver during an inflammatory response, such as during cytomegalovirus induced hepatitis.^241,242^

One of our recent studies revealed that a gene encoding a well-known m^6^A reader, YTHDF2, positively controlled NK cell egress from the BM to the periphery. Knocking out YTHDF2 from NK cells significantly reduced NK cell numbers in peripheral blood but not in the BM. Through i.v. injection of anti-CD45 antibodies to label immune cells, we found a significantly lower frequency of CD45^+^ YTHDF2-deficient NK cells than wild-type NK cells in the sinusoids of the BM.^243^ However, the detailed mechanisms that allow YTHDF2 to regulate NK cell trafficking are still largely unknown.

### Cell-extrinsic factors affecting NK cell homing and trafficking

#### Cytokines

Several cytokines, such as IL-2, IL-12, and IL-15, are critical for NK cell development, survival, and activation, and they are also used to expand NK cells before adoptive transfer. Cytokine treatments have profound and diverse effects on the chemokine receptor profiles of NK cells, depending on dosage and length of treatment (Fig. 5).

The preferred concentration of IL-2 for expanding NK cells in the clinic is ~1000 IU/ml. After NK cells were treated with that dose for 48 h, both CXCR1 and CXCR4 were downregulated, while CXCR3 was upregulated. Consequently, the cells showed reduced homing to the BM and instead trafficked into inflammatory sites, which could limit their alloreactive potential against hematopoietic malignancies.^244^ A 6-day treatment with 100 IU/ml IL-2 increased CXCR6 expression on human peripheral NK cells.^245^ After NK-92 cells or human primary NK cells were incubated with IL-2 (100 U/ml) for 3 to 24 h, CXCR3 mRNA was rapidly downregulated whereas both *CCR1* and *CXCR4* mRNA were strongly upregulated.^246^ However, the CXCR4 data differed if the cells were treated with 1000 IU/ml IL-2 for 48 h.^244^

IL-12 is one of the stimulators that promote the production of IFN-γ, which also cooperates with IL-18 to enhance the cytotoxicity of NK cells.^247^ After NK cells were stimulated with IL-12 for just 3 h, expression of CCR1, CCR2, and CXCR4 was upregulated while expression of CXCR3 is downregulated.^246^ Stimulation of NK cells with IL-12 for over 24 h also downregulated the expression of CXCR3 and upregulated the expression of CX3CR1.^246^ In early studies, stimulation with IL-12 increased the binding of human NK cells to endothelial cells. Mechanistically, IL-12 affects the activity of LFA-1 molecules and enhances the chemotactic response of NK cells.^248^ These studies suggest that expression of chemokine receptors can be modulated for therapeutic purposes by culturing NK cells ex vivo. To attack tumor cells in the BM, for example, NK cells could first be expanded with 1000 IU/ml IL-2. Then the expanded population can be stimulated for a few hours with IL-12 to upregulate CXCR4 and downregulate CXCR3 before adoptive transfer. This strategy might help NK cells home back into the BM more efficiently.

IL-15, a pivotal cytokine for NK cell development and survival,^234,249^ strongly represses mRNA and protein levels of CX3CR1. In contrast, IL-2 has the opposite effect.^250^ After BM cells were stimulated with IL-15 (100 ng/ml) for 10 days, CX3CR1 expression was almost undetectable.^250^ Such low levels reduced the chemotaxis of NK cells to CX3CL1 in mice.^251^ In cultured CD56^+^ NK cells, short-term stimulation with IL-15 decreased surface expression of CX3CR1 and diminished the cells’ chemotaxis toward CX3CL1. After long-term co-culture with IL-15 (>5 days), NK cells showed reduced expression of CD62L and CXCR1, undetectable mRNA and protein expression of CX3CR1, and increased expression of CXCR3 and CXCR6.^252^ However, long-term stimulation with IL-15 did not affect the expression of CXCR4 or the cells’ responsiveness to CXCL12, a CXCR4 ligand, in mice^250^ and humans.^251^

Stimulation with combinations of the above interleukins dramatically changes chemokine receptor expression profiles in a complex manner. After NK-92 cells or human primary NK cells were incubated with IL-2 (100 U/ml) in combination with IL-12 (10 U/ml) or IL-18 (10 ng/ml) for 3–24 h, *CXCR3* mRNA was rapidly downregulated. In contrast, both *CCR1* and *CXCR4* mRNA were strongly upregulated.^246^ Moreover, treatment with 10 ng/ml IL-12 plus 25 ng/ml IL-15 for 6 days increased the expression of CXCR6 on human peripheral blood NK cells but not on hepatic NK cells. Stimulation with 100 IU/ml IL-2 plus 25 ng/ml IL-15 for 20 h upregulated the expression of CXCR6 on human peripheral blood NK cells.^245^ IL-18 is often used in combination with other cytokines, but by itself it helps upregulate CCR7 in NK cells.^253,254^

Some other cytokines are also reported to regulate NK cell migration. NK cells exert their anti-tumor effects by producing IFN-γ, which is also required to recruit NK cells into the TME. In mice, IFN-γ promoted CXCR3 expression on NK cells and also enhanced the production of its ligands, CXCL9 and CXCL10, on virus-infected cells and tumor cells;^183,255^ these ligands are important for NK cells’ anti-infection and anti-tumor properties. In a mouse model of IFN-γ-induced abortion, IFN-γ increased the expression of CX3CL1 in the uterus, prompting CD49b^+^ NK cells to home into the endometrium and eventually cause pregnancy failure.^118^

In healthy human donors, in vivo administration of granulocyte colony-stimulating factor (G-CSF) enhanced the expression of CD62L and CXCR4 on the surface of NK cells in peripheral blood.^256^ TGF-β, an important immunosuppressive factor in the TME, increased the expression of CXCR4, CX3CR1, and CXCR3 on NK cells in vitro.^257^ This enhancement may potentially promote the egress of NK cells from the TME and represents a novel mechanism for tumor escape. Blocking TGF-β signaling increased allogeneic NK cells against glioblastoma stem cells in a glioblastoma stem cell-engrafted mice model.^258^ Whether blocking TGF-β affects the infiltration of NK cells was not addressed in that study.

Despite the lack of evidence that cytokines directly affect NK cell homing and trafficking in vivo, cytokines alone or in a cocktail with other cytokines extensively change chemokine receptor profiles in vitro. Thus, it is pivotal to establish a precise protocol for expanding NK cells in vitro, especially by specifying the types and concentrations of cytokines that would be appropriate for a particular type of cancer.

#### Hypoxia in the TME

Hypoxia, a common feature of tumors, profoundly influences the expression patterns of chemokines, cytokines, and chemokine receptors of NK cells. It also affects the cells’ chemotaxis toward specific chemokines, such as CCL19, CCL21, and CXCL12.^259^ When cultured under hypoxic conditions in vitro for 24 to 96 h, peripheral blood NK cells showed increased surface expression of CXCR4 and CCR7; this effect occurred preferentially (CXCR4) or selectively (CCR7) in CD56^bright^ NK cells (Fig. 5).^259^ Similar phenotypic changes, such as downregulation of CCR1, CCR5, and CXCR3, were observed in the TME in vivo.^173^

#### Low pH in the TME

Owing to lack of oxygen, tumor cells must obtain energy through anaerobic glycolysis, which leads to the accumulation of lactic acid. At the same time, ion exchange proteins on the plasma membrane continue to transport H^+^ from the cell interior to its exterior to avoid tumor cell self-acidosis. These cellular reactions decrease the TME’s pH, leading to various levels of acidity. Studies have shown that tumor-derived lactic acid increases the number of myeloid-derived suppressor cells, which reduces the cytotoxicity of NK cells. Thus, tumor cells indirectly inhibit the functionality of NK cells by producing lactic acid.^260^ In an immunogenic mouse model of melanoma, tumors that produced less lactic acid developed at a significantly slower rate than the control group, and a greater number of IFN-γ^+^ NK cells infiltrated the tumor sites.^261^ This indicates that lactic acid may also inhibit the invasion of NK cells into the TME.

#### High levels of adenosine in the TME

Adenosine also promotes the accumulation of tumor-derived metabolites.^262^ Adenosine monophosphate inhibits the maturation of NK cells in the TME, mainly via the adenosine A2A receptor. When that receptor was knocked out, CX3CR1 transcription was upregulated in NK cells, suggesting that adenosine monophosphate may inhibit the infiltration of NK cells into the TME by inhibiting their expression of CX3CR1 (Fig. 5).^263^

### Intercellular interactions

Interactions among cells also help regulate NK cell trafficking (Fig. 5). Therefore, understanding the effects of various cells on the chemotaxis of NK cells is essential for understanding NK cell dynamics in the TME. DCs are a main orchestrator of anti-tumor immune responses, and the effects of their interactions with NK cells in the TME on anti-tumor immunity have been extensively reviewed.^156,264^ DCs can activate NK cells by producing IL-12, IL-15, and IL-18, thus enhancing anti-tumor activity.^265–267^ Generating CXCL9 and CXCL10 to recruit effector T cells is also part of DCs’ anti-tumor repertoire.^268^ However, whether CXCL9 and CXCL10 produced by DCs have the same effect on NK cells during disease states needs to be verified. After intraperitoneal injection of plasmacytoid DC in mice, the number of NK cells in the abdominal cavity increased significantly. This process depended on the expression of CXCR3 and CD62L in NK cells.^269^

T cells, the most important contributors to adaptive immunity, are classified as CD4 (CD4^+^ or helper T cells) or CD8 (CD8^+^ or cytotoxic T cells) according to their functional characteristics. In a mouse tumor model, NK cells activated at a distant site by a TLR7/8 agonist released tumor antigens and induced tumor-specific CD4^+^ T cells. However, depleting CD4^+^ T cells significantly reduced the number of NK cells in the TME in that model, indicating that CD4^+^ T cells actively recruit NK cells to the tumor site. Mechanistically, CD4^+^ cells in the tumor recruits NK cells to the tumor site by stimulating tumor cells and other immune-infiltrating cells to release CXCL10.^270^ CD8^+^ T cells and NK cells are both cytolytic lymphocytes. At present, it is known that CD8^+^ T cells can promote the maturation of liver-resident NK cells,^271^ but there is little information regarding the regulation of NK cells by CD8^+^ T cells in tumors.

In African green monkeys infected with simian immunodeficiency virus (SIV), IL-6 produced by B cells induced NK cells to express CXCR5 and migrate to B-cell follicles to combat the virus.^272^ This suggests that B cells regulate NK cell trafficking via CXCR5.

In an LPS-induced acute lung injury mouse model, T-bet^+^ NK cells were the critical source of pulmonary CXCL1 and CXCL2, contributing to the recruitment of polymorphonuclear neutrophils.^273^ This might indicate crosstalk between NK cells and neutrophils.

In a mouse osteoarthritis model, NK cells and neutrophils were among the first cells to accumulate in the synovium of the joint capsule and promote the progression of osteoarthritis. Depletion of neutrophils reduced the number of NK cells in the synovium, revealing that neutrophils are responsible for recruiting NK cells to the synovium, likely by expressing CXCL10.^274^

To sum up, changes in environmental factors can also affect the migration of NK cells into the TME. Thus, improving the microenvironment might be a strategy for enhancing the infiltration of NK cells into diseased tissue.

## Strategies to improve CAR NK cell efficiency to enhance NK celll trafficking

### Historical progress with CAR NK cells

CAR is a synthetic cell-surface receptor that redirects T cells, NK cells, NKT cells, γδT cells, and macrophages toward tumor cells carrying the corresponding antigens, and all of those cells show promise for improving tumor immunotherapy.^275–280^ CAR proteins have an extracellular antigen-recognition domain and multiple intracellular signal activation domains that dramatically activate intracellular immune responses after they bind to tumor antigens.^281^ CAR T cell therapy has enjoyed unprecedented progress in hematological diseases, and it has already been approved by the US Food and Drug Administration for treating some hematological malignancies.^282–284^ Other CAR immune cell therapies are summarized in Table 2.^275–280^ The big advantage of CAR NK cells is that NK cells can be used without human leukocyte antigen matching; thus, allogeneic CAR NK cells are efficacious against tumor cells without producing obvious graft versus-host disease (GVHD) or a cytokine storm.^12,14,29^ For this reason, CAR NK cells have the potential to become “off-the-shelf” anti-cancer immunotherapeutic products.^29^ Owing to their recent great success in clinical trials against hematological malignancies,^12,14,29^ CAR NK cells are attracting more attention than ever.

**Table 2:** Comparison of different kinds of CAR immune cells

	Cell sources	Features	Cytotoxicity mechanisms	Cytokine release syndrome and neurotoxicity	Clinical trials
CAR NK	Autologous, non-MHC-matched allogeneic peripheral or umbilical cord blood derived, cell lines, and iPSC-derived	Multiple killing mechanisms; rapid cytotoxicity; no antigen priming needed	Both CAR-dependent and CAR-independent cell killing	Less common	Rich clinical trials
CAR T	Autologous, MHC-matched allogeneic, iPSC-derived	MHC independent TCR; sufficient sources; high specificity	CAR-dependent cell killing	Common	FDA-approved; Rich clinical trials
CAR NKT cells	Autologous, iPSC-derived	Innate and adaptive features; TCR recognizes lipid antigens presented by CD1d	CAR-dependent cell killing; killing of target cells by endogenous TCR	No clinical data, but expected to be common	Fewer clinical trials
CAR γδT	Autologous, iPSC-derived	MHC independent γδTCR; cross presentation of antigens to γδT cells	CAR-dependent cell killing; CAR-independent γδT-mediated cell killing	No clinical data, but expected to be common	Fewer clinical trials
CAR Macrophages	Autologous, iPSC-derived, cell lines	Phagocytosis signaling domains; better at penetrate into solid tumors	CAR-dependent phagocytosis; macrophage-mediated immunostimulatory TME; macrophage-mediated alteration of TME; macrophages as antigen-presenting cells to stimulate immune response	No clinical data, but expected to be common	Only one clinical trial

**Fig. 6:**
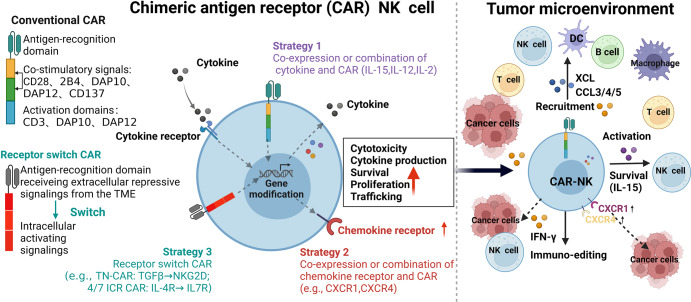
Strategies to improve the infiltration, activation, and survival of CAR-NK cells in the TME. Expressing a CAR on NK cells can alter the profiles of chemokine receptors, selectins, and cytokines, boosting the infiltration of NK cells into the TME and modulating the TME’s immune microenvironment. Co-expression and administration of cytokine(s) and chemokine(s) or a corresponding receptor with a CAR have multiple effects on NK cell-based immunotherapy, including on infiltration, activation, and survival of NK cells in the TME. In addition, a switch receptor strategy was used to equip a CAR with an extracellular domain targeting a suppressive antigen in the TME and an intracellular domain with an activation signal. This strategy (to produce TN-CAR and 4/7 ICR CAR, for example) converts the TME’s suppressive signal into an activation signal, enhancing NK cell effector functions. 4/7 ICR-CAR: IL4R-IL-7R-CAR. Created with BioRender.com

Many preclinical studies have revealed that genetic modification of intrinsic and extrinsic inhibiting or activating pathways can improve the functioning and persistence of CAR NK cells in the TME of solid tumors, indicating their potential against non-hematological tumors.^285^ In fact, several preclinical trials have used CAR NK cells to treat solid tumors by targeting NKG2D ligands, Roundabout homolog 1 (ROBO1), HER2, Mesothelin, Prostate-specific membrane antigen (PSMA), and Mucin 1 (MUC1) with surprising results.^29^ We recently developed a CAR NK cell directed against prostate stem cell antigen (PSCA), which showed impressive therapeutic efficiency in human metastatic pancreatic cancer models.^231^ A new source of NK cells—iPSC-derived NK cells (iPSC-NKs)—has also shown more promise with solid tumors (e.g., ovarian cancer) than CAR T cells or peripheral blood cell-derived CAR NK cells.^26^ Therefore, many clinical trials have started to test the possibility of using CAR NK cells in both hematological and solid tumors including ovarian cancer (NCT03692637), prostate cancer (NCT03692663), pancreatic cancer (NCT03941457), and solid tumors to target NKG2D-L (NCT03415100, NCT03940820, NCT05213195). CAR NK use is not limited to tumor therapy, however. We recently generated ACE2-CAR NK cells that target the spike protein of SARS-CoV-2, and tested them in the K18-hACE2 animal model.^286^ Also, NK cells co-expressing NKG2D- and ACE2-CAR have been designed to treat patients with COVID-19, an infectious disease caused by SARS-CoV-2 (NCT04324996). For details of clinical trials, please see Table 3. However, the therapeutic efficiency of CAR NK cells sometimes is less than desired. Therefore, in this review, we focus mainly on how co-expressing CAR and chemotactic receptor affects NK cell trafficking (Summarized in Fig. 6).

**Table 3:** Ongoing clinical trials related to CAR-NK cells

CAR-name	Condition or disease	Intervention/treatment	Phase	Identifier
HLA haploidentical CD19-CAR NK cells	B-cell NHL	/	Phase 1	NCT04887012
CD19-CAR NK with IL-15R fusion, CD19-CAR NK cells expressing IL-15	BCL, CLL, R/R NHL	Cyclophosphamide, Fludarabine, Mesna	Phase 1/2	NCT04639739 NCT03056339
CD19-CAR-IL-15-transduced cord blood NK cells	BCL	Rituximab, Carmustine, Cytarabine, Etoposide	Phase 1/2	NCT03579927
BCMA-CAR NK92 cells	MM	Fludarabine, Cytoxan (Cyclophosphamide)	Phase 1/2	NCT03940833 NCT05008536
CD33/CLL1-CAR NK cells	AML	Fludarabine, Cytoxan (Cyclophosphamide)	Phase 1	NCT05215015 NCT05008575
CD33-CAR NK cells	AML	/	Phase 1/2	NCT02944162
CD22-CAR NK cells	R/R BCL	/	Early Phase 1	NCT03692767
FT596: iPSC-CAR-19 NK cells, IL-15R fusion	BCL, CLL, NHL	Cyclophosphamide, Fludarabine, Rituximab, Obinutuzumab	Phase 1	NCT04245722 NCT04555811
FT576: iPSC-BCMA-CAR NK cells, IL-15R fusion	MM	Cyclophosphamide, Fludarabine, Daratumumab	Phase 1	NCT05182073
CD5-CAR NK cells, IL-15 fusion	Hematological malignancies	Fludarabine phosphate, Cyclophosphamide	Phase 1/2	NCT05110742
PDL1-CAR NK cells, IL-2 fusion	Various	Nivolumab, Atezolizumab, Avelumab, Pembrolizumab, Durvalumab	Phase 2	NCT04390399 NCT03228667 NCT04847466
NKG2D-ACE2-CAR NK cells, IL-15 fusion	COVID-19	IL-15-NK cells	Phase 1/2	NCT04324996
NKG2D-CAR NK cells	AML, MDS, refractory metastatic colorectal cancer, metastatic solid tumors	/	Phase 1	NCT04623944 NCT05213195 NCT05247957 NCT03415100
HER2-CAR NK cells	Glioblastoma	/	Phase 1	NCT03383978
5T4-CAR NK cells	5T4-positive advanced solid tumors	/	Early Phase 1	NCT05194709
Mesothelin-CAR NK cells	Epithelial ovarian cancer	/	Early phase 1	NCT03692637
PSMA-CAR NK cells	Castration-resistant prostate cancer	/	Early phase 1	NCT03692663
ROBO1-CAR NK cells	ROBO1-positive tumors/Pancreatic cancer	/	Phase 1/2	NCT03940820 NCT03931720 NCT03941457

*R*/*R* relapsed/refractory, *BCL* B-cell lymphoma, *BCMA* B-cell mature antigen, *Mesna* 2-mercaptoethane sulfonate sodium

### Strategies for improving infiltration and effector functions of CAR NK cells

CAR T cells produce abundant TNF-α, IL-1β, and IL-6, which can cause cytokine release syndrome when tumor cells are targeted with specific antigens. In contrast, CAR NK cells launch anti-tumor activity via direct cytotoxicity, as they release neoantigens after lysing target cells, and the abundant cytokines they produce, especially IFN-γ, do not trigger cytokine release syndrome.^278^ IFN-γ, a strong immune enhancer, plays multifaceted roles in remodeling the TME. As well as directly inhibiting tumor proliferation and targeting cancer stem-like cells, it also increases MHC expression and antigen presentation, thereby augmenting the functions of tumor-infiltrating adaptive immune cells including Th1 cells and cytotoxic T lymphocytes (CTLs). IFN-γ also suppresses Treg cell function and angiogenesis mediated by stromal cells.^157,287^ Thus, strategies for enhancing the infiltration of CAR NK cells in the TME could play many roles in improving treatment outcomes.

There have been intense efforts to infiltrate enough CAR cells into tumors to overcome the obstacles in the hostile TME. In several preclinical studies, co-expression of a CAR and a chemokine receptor on T cells increased the cells’ invasion into tumors and also boosted their effectiveness against solid tumors.^288^ CAR T cells equipped with CXCR2,^289^ CCR2b,^290^ or CXCR5^291^ increased their chemotaxis to tumor cells, where the anti-tumor effects of T cells are enhanced. Overexpression of CXCR1 did not increase the cytotoxicity of NKG2D-CAR NK cells, but it boosted trafficking into tumors to reduce tumor load after ovarian cancer cells were injected intravenously or peritoneally into mice.^292^ Transgenic expression of CXCR4 on human CD19-CAR NK92 cells or human primary NK cells also increased chemotaxis to BM stromal cells without affecting the cells’ CD19-specific cytotoxic activity.^69^ EGFR-CAR NK cells with CXCR4 overexpression showed increased chemotaxis toward U87-MG glioblastoma cells that secreted CXCL12/SDF-1α, improving survival in a mouse model of glioblastoma.^293^ Co-expression of CCR7 with high-affinity CD16 improved rituximab-induced ADCC against lymphoma cells, K562 cells, or MM.1 S cells. It also increased NK cell migration toward the lymph node-associated chemokine CCL19 in vitro.^294^

In addition to combining targeted chemokine receptors with CAR NK cells, co-expressing or combining cytokines or chemokines with CAR NK cells may play a role in cancer treatment. Co-expressed IL-15 and CD19-CAR NK cells showed long-term persistence and expansion as well as the stronger anti-tumor activity in vivo.^295^ Co-expression of NKG2D-CAR and IL-15 in the PiggyBac system significantly increased the anti-AML activity of human peripheral blood NK cells.^296^ In the latest study, PSCA-CAR NK cells expressing soluble IL-15 showed therapeutic efficacy in human metastatic pancreatic cancer models without signs of systematic toxicity, providing a strong rationale for clinical development.^231^ Owing to the increased understanding the structure bases of the pleiotropy of cytokine receptors and the great advance of synthetic biology, engineered cytokines or chemokines improve their specificity or affinity to target cells and reduce their side effects. For example, engineered IL-2 “superkine”, IL-15 “superagonist”, and IL-12 partial agonists show enhanced affinity or specificity to target cells, including NK cells.^297^ Thus, in the future, the combination of CAR NK cells and cytokines or chemokines should also be more extensively tested in the hope of acquiring a desired therapeutic effect.

To overcome the suppressive TME caused by TGF-β, IL-4, and IDO, a switch receptor strategy was recently developed. It equipped a CAR with an extracellular domain targeting suppressive antigen in the TME and an intracellular domain with activation signals.^278^ One a CAR was designed to fuse the extracellular and transmembrane domains of the TGF-β type II receptor with the intracellular domain of NKG2D, referred to as TN-CAR (T for TGF-β and N for NKG2D). When NK-92 cells engineered with TN-CAR encounter tumor cells that express TGF-β, the intracellular domain of NKG2D converts the immunosuppressive signaling of TGF-β into an activating signal. Thus, TN-CAR NK-92 cells show increased chemotaxis toward tumor cells expressing TGF-β. They also repressed the differentiation of naïve CD4^+^ T cells into regulatory T cells in vitro.^298^ In a xenograft tumor model of HCC, TN-CAR NK-92 cells reduced tumor load and show increased infiltration into tumor because TN-CAR increased the surface expression of CCR3, CCR6, CXCR4, and CX3CR1 on the NK-92 cells.^298^ This evidence suggests that CAR expression may affect the profiles of chemokine receptors on NK cells, which should be considered when deciding which types of cancer to target in vivo. A similar switch receptor strategy was used to design a CAR construct that converts IL-4-mediated suppressive signals into proliferative signals transmitted by IL-7. This 4/7 inverted cytokine receptor (4/7 ICR) has both the ectodomain of the IL-4 receptor and the endodomain of the IL-7 receptor. Combining 4/7 ICR and CAR-PSCA in T cells enhanced anti-tumor activity against PSCA^+^ pancreatic cancer, which is characterized by a dense immunosuppressive environment rich in IL-4.^299^ These investigations suggest that the switch receptor strategy will be useful in the future for improving the therapeutic efficiency of CAR NK cells.

In summary, genetically modifying CAR NK cells to enhance their chemotaxis to tumor cells and overcome immunosuppressive obstacles in the TME appears to be a hopeful strategy for using such cells against cancer in the near future. However, most of the above studies were performed in rodent models, especially mice, due to low animal cost, low drug consumption, short growth cycle, and wide availability of transgenic animals.^300^ However, the murine models may not recapitulate humans as the two species may have different trafficking molecules.^58^ Therefore, therapies that are effective in mice might not necessarily be effective in humans.

## Conclusion and future perspectives

NK cell-based immunotherapy, especially with CAR NK cells, has proven safe and effective. It also has the potential to produce allogeneic, “off-the-shelf” products and to benefit even relapsed cancer patients, including some who are resistant to CAR T cell therapies. Increased understanding of the mechanisms that regulate NK cell homing and trafficking in various normal tissues and the TME will provide us with more strategies for improving the infiltration and anti-tumor immunity of both endogenous and exogenous NK cells. Altering the expression levels of chemokines or integrin ligands in the TME will help enhance the infiltration, expansion, and activation of endogenous NK cells in the TME. With improved methods for expanding NK cells expansion in vitro and better understanding of how in vitro expansion affects the chemokine receptor profiles of NK cells, it will become possible to further enhance the therapeutic efficiency of NK cell adoptive transfer (e.g., CAR NK, CAR iPSC-NK cells) by using gene editing, optimizing in vitro expansion protocols, and combining NK cells with other treatment(s) (e.g., cytokines, chemokines, or antibodies) to modulate NK cell trafficking. Via gene editing or in vitro training, we could also endow NK cells with the capacity to immunomodulate the TME from “cold” to “hot” to arouse exhausted adaptive immune cells into a collective fight against cancer. One experimental study demonstrated that NKG2D-CAR NK cells can kill myeloid-derived suppressor cells in the TME, which would help improve the activity of CAR T cells against solid tumors.^301^ Also, each type of CAR immune cell therapy has its own advantages and limitations, and different CAR cells can help each other in the TME. In most cases, moreover, combination therapies are usually more effective than monotherapies. Thus, we should combine CAR NK cells with other therapeutics, including another cell therapy such as CAR T cells or CAR NK cells that target a different antigen, to have optimal efficacy in treating cancer, especially solid tumors.

## Data Availability

Not applicable.

## References

[1] Scoville SD, Freud AG, Caligiuri MA (2017). Modeling human natural killer cell development in the era of innate lymphoid cells. Front. Immunol..

[2] Stokic-Trtica V, Diefenbach A, Klose CSN (2020). NK cell development in times of innate lymphoid cell diversity. Front. Immunol..

[3] Vivier E (2018). Innate lymphoid cells: 10 years on. Cell.

[4] Freud AG, Mundy-Bosse BL, Yu J, Caligiuri MA (2017). The broad spectrum of human natural killer cell diversity. Immunity.

[5] Bjorkstrom NK, Ljunggren HG, Michaelsson J (2016). Emerging insights into natural killer cells in human peripheral tissues. Nat. Rev. Immunol..

[6] Yu J, Freud AG, Caligiuri MA (2013). Location and cellular stages of natural killer cell development. Trends Immunol..

[7] Vivier E, Tomasello E, Baratin M, Walzer T, Ugolini S (2008). Functions of natural killer cells. Nat. Immunol..

[8] Fang F, Xiao W, Tian Z (2017). NK cell-based immunotherapy for cancer. Semin. Immunol..

[9] Hammer Q, Ruckert T, Romagnani C (2018). Natural killer cell specificity for viral infections. Nat. Immunol..

[10] Bjorkstrom NK, Strunz B, Ljunggren HG (2022). Natural killer cells in antiviral immunity. Nat. Rev. Immunol..

[11] Li Y (2021). Natural killer cells: friend or foe in metabolic diseases?. Front. Immunol..

[12] Shimasaki N, Jain A, Campana D (2020). NK cells for cancer immunotherapy. Nat. Rev. Drug Discov..

[13] Huntington ND, Cursons J, Rautela J (2020). The cancer-natural killer cell immunity cycle. Nat. Rev. Cancer.

[14] Maskalenko, N. A., Zhigarev, D. & Campbell, K. S. Harnessing natural killer cells for cancer immunotherapy: dispatching the first responders. *Nat. Rev. Drug Discov.* (2022). 10.1038/s41573-022-00413-7 PMC10019065 35314852

[15] Pockley AG, Vaupel P, Multhoff G (2020). NK cell-based therapeutics for lung cancer. Expert Opin. Biol. Ther..

[16] Muntasell A (2019). NK cell infiltrates and HLA class I expression in primary HER2(+) breast cancer predict and uncouple pathological response and disease-free survival. Clin. Cancer Res..

[17] Liu P, Chen L, Zhang H (2018). Natural killer cells in liver disease and hepatocellular carcinoma and the NK cell-based immunotherapy. J. Immunol. Res..

[18] Ali TH (2014). Enrichment of CD56(dim)KIR + CD57 + highly cytotoxic NK cells in tumour-infiltrated lymph nodes of melanoma patients. Nat. Commun..

[19] Terren I (2020). NK cell-based immunotherapy in renal cell carcinoma. Cancers (Basel).

[20] Rosenberg SA (1985). Observations on the systemic administration of autologous lymphokine-activated killer cells and recombinant interleukin-2 to patients with metastatic cancer. N. Engl. J. Med..

[21] Rosenberg SA (1987). A progress report on the treatment of 157 patients with advanced cancer using lymphokine-activated killer cells and interleukin-2 or high-dose interleukin-2 alone. N. Engl. J. Med..

[22] Tran AC, Zhang D, Byrn R, Roberts MR (1995). Chimeric zeta-receptors direct human natural killer (NK) effector function to permit killing of NK-resistant tumor cells and HIV-infected T lymphocytes. J. Immunol..

[23] Ruggeri L (2002). Effectiveness of donor natural killer cell alloreactivity in mismatched hematopoietic transplants. Science.

[24] Miller JS (2005). Successful adoptive transfer and in vivo expansion of human haploidentical NK cells in patients with cancer. Blood.

[25] Tang X (2018). First-in-man clinical trial of CAR NK-92 cells: safety test of CD33-CAR NK-92 cells in patients with relapsed and refractory acute myeloid leukemia. Am. J. Cancer Res..

[26] Li Y, Hermanson DL, Moriarity BS, Kaufman DS (2018). Human iPSC-derived natural killer cells engineered with chimeric antigen receptors enhance anti-tumor activity. Cell Stem Cell.

[27] Goldenson BH, Kaufman DS (2021). Into the multiverse of gene edited NK cell-based therapeutic strategies. Cell Stem Cell.

[28] Liu E (2020). Use of CAR-transduced natural killer cells in CD19-positive lymphoid tumors. N. Engl. J. Med..

[29] Yilmaz A, Cui H, Caligiuri MA, Yu J (2020). Chimeric antigen receptor-engineered natural killer cells for cancer immunotherapy. J. Hematol. Oncol..

[30] Riggan L, Shah S, O’Sullivan TE (2021). Arrested development: suppression of NK cell function in the tumor microenvironment. Clin. Transl. Immunol..

[31] Hu Z, Xu X, Wei H (2021). The adverse impact of tumor microenvironment on NK-Cell. Front. Immunol..

[32] Melaiu O, Lucarini V, Cifaldi L, Fruci D (2019). Influence of the tumor microenvironment on NK cell function in solid tumors. Front. Immunol..

[33] Gauthier L (2019). Multifunctional natural killer cell engagers targeting NKp46 trigger protective tumor immunity. Cell.

[34] Scoville SD, Freud AG, Caligiuri MA (2019). Cellular pathways in the development of human and murine innate lymphoid cells. Curr. Opin. Immunol..

[35] Rosmaraki EE (2001). Identification of committed NK cell progenitors in adult murine bone marrow. Eur. J. Immunol..

[36] Bai L (2021). Liver type 1 innate lymphoid cells develop locally via an interferon-gamma-dependent loop. Science.

[37] Montaldo E, Vacca P, Moretta L, Mingari MC (2014). Development of human natural killer cells and other innate lymphoid cells. Semin. Immunol..

[38] Kondo M, Weissman IL, Akashi K (1997). Identification of clonogenic common lymphoid progenitors in mouse bone marrow. Cell.

[39] Morrison SJ, Weissman IL (1994). The long-term repopulating subset of hematopoietic stem cells is deterministic and isolatable by phenotype. Immunity.

[40] Fathman JW (2011). Identification of the earliest natural killer cell-committed progenitor in murine bone marrow. Blood.

[41] Cui G (2014). Characterization of the IL-15 niche in primary and secondary lymphoid organs in vivo. Proc. Natl Acad. Sci. USA.

[42] Ma S, Caligiuri MA, Yu J (2022). A four-stage model for murine naturlal killer cell development in vivo. J. Hematol. Oncol..

[43] Abel AM, Yang C, Thakar MS, Malarkannan S (2018). Natural killer cells: development, maturation, and clinical utilization. Front. Immunol..

[44] Chiossone L (2009). Maturation of mouse NK cells is a 4-stage developmental program. Blood.

[45] Hayakawa Y, Smyth MJ (2006). CD27 dissects mature NK cells into two subsets with distinct responsiveness and migratory capacity. J. Immunol..

[46] Boudreau JE, Hsu KC (2018). Natural killer cell education and the response to infection and cancer therapy: stay tuned. Trends Immunol..

[47] Freud AG, Yu J, Caligiuri MA (2014). Human natural killer cell development in secondary lymphoid tissues. Semin. Immunol..

[48] Cichocki F, Grzywacz B, Miller JS (2019). Human NK cell development: one road or many?. Front. Immunol..

[49] Cooper MA (2001). Human natural killer cells: a unique innate immunoregulatory role for the CD56(bright) subset. Blood.

[50] Yu J (2010). CD94 surface density identifies a functional intermediary between the CD56bright and CD56dim human NK-cell subsets. Blood.

[51] Romagnani C (2007). CD56brightCD16- killer Ig-like receptor- NK cells display longer telomeres and acquire features of CD56dim NK cells upon activation. J. Immunol..

[52] Milush JM (2009). Functionally distinct subsets of human NK cells and monocyte/DC-like cells identified by coexpression of CD56, CD7, and CD4. Blood.

[53] Carrega P (2014). CD56(bright)perforin(low) noncytotoxic human NK cells are abundant in both healthy and neoplastic solid tissues and recirculate to secondary lymphoid organs via afferent lymph. J. Immunol..

[54] Grzywacz B (2011). Natural killer-cell differentiation by myeloid progenitors. Blood.

[55] Dege C (2020). Potently cytotoxic natural killer cells initially emerge from erythro-myeloid progenitors during mammalian development. Dev. Cell.

[56] Wu C (2014). Clonal tracking of rhesus macaque hematopoiesis highlights a distinct lineage origin for natural killer cells. Cell Stem Cell.

[57] Wu C (2018). Clonal expansion and compartmentalized maintenance of rhesus macaque NK cell subsets. Sci. Immunol..

[58] Sternberg-Simon M (2013). Natural killer cell inhibitory receptor expression in humans and mice: a closer look. Front. Immunol..

[59] Peng H, Tian Z (2014). NK cell trafficking in health and autoimmunity:a comprehensive review. Clin. Rev. Allergy Immunol..

[60] Yao X, Matosevic S (2021). Chemokine networks modulating natural killer cell trafficking to solid tumors. Cytokine Growth Factor Rev..

[61] Shannon MJ, Mace EM (2021). Natural killer cell integrins and their functions in tissue residency. Front. Immunol..

[62] van Spriel AB (2001). Mac-1 (CD11b/CD18) is essential for Fc receptor-mediated neutrophil cytotoxicity and immunologic synapse formation. Blood.

[63] Mace EM, Zhang J, Siminovitch KA, Takei F (2010). Elucidation of the integrin LFA-1-mediated signaling pathway of actin polarization in natural killer cells. Blood.

[64] Mace EM, Monkley SJ, Critchley DR, Takei F (2009). A dual role for talin in NK cell cytotoxicity: activation of LFA-1-mediated cell adhesion and polarization of NK cells. J. Immunol..

[65] Mayol K, Biajoux V, Marvel J, Balabanian K, Walzer T (2011). Sequential desensitization of CXCR4 and S1P5 controls natural killer cell trafficking. Blood.

[66] Bernardini G (2008). CCL3 and CXCL12 regulate trafficking of mouse bone marrow NK cell subsets. Blood.

[67] Broxmeyer HE (2005). Rapid mobilization of murine and human hematopoietic stem and progenitor cells with AMD3100, a CXCR4 antagonist. J. Exp. Med..

[68] Inngjerdingen M, Damaj B, Maghazachi AA (2001). Expression and regulation of chemokine receptors in human natural killer cells. Blood.

[69] Jamali A (2020). Highly efficient generation of transgenically augmented CAR NK cells overexpressing CXCR4. Front. Immunol..

[70] Levy E (2019). Enhanced bone marrow homing of natural killer cells following mRNA transfection with gain-of-function variant CXCR4(R334X). Front. Immunol..

[71] Sciume G (2011). CX3CR1 expression defines 2 KLRG1+ mouse NK-cell subsets with distinct functional properties and positioning in the bone marrow. Blood.

[72] Ponzetta A (2013). CX3CR1 regulates the maintenance of KLRG1+ NK cells into the bone marrow by promoting their entry into circulation. J. Immunol..

[73] Yang C (2019). Heterogeneity of human bone marrow and blood natural killer cells defined by single-cell transcriptome. Nat. Commun..

[74] Seo S, Mace EM (2022). Diversity of human NK cell developmental pathways defined by single-cell analyses. Curr. Opin. Immunol..

[75] Pereira JP, An J, Xu Y, Huang Y, Cyster JG (2009). Cannabinoid receptor 2 mediates the retention of immature B cells in bone marrow sinusoids. Nat. Immunol..

[76] Mazo IB (1998). Hematopoietic progenitor cell rolling in bone marrow microvessels: parallel contributions by endothelial selectins and vascular cell adhesion molecule 1. J. Exp. Med..

[77] Plavina T (2017). Reversibility of the effects of natalizumab on peripheral immune cell dynamics in MS patients. Neurology.

[78] Mellergard J (2013). Increased B cell and cytotoxic NK cell proportions and increased T cell responsiveness in blood of natalizumab-treated multiple sclerosis patients. PLoS ONE.

[79] Jiang D (2004). Regulation of pulmonary fibrosis by chemokine receptor CXCR3. J. Clin. Invest..

[80] Bonanni V, Antonangeli F, Santoni A, Bernardini G (2019). Targeting of CXCR3 improves anti-myeloma efficacy of adoptively transferred activated natural killer cells. J. Immunother. Cancer.

[81] Wald O (2006). IFN-gamma acts on T cells to induce NK cell mobilization and accumulation in target organs. J. Immunol..

[82] Walzer T (2007). Natural killer cell trafficking in vivo requires a dedicated sphingosine 1-phosphate receptor. Nat. Immunol..

[83] Jenne CN (2009). T-bet-dependent S1P5 expression in NK cells promotes egress from lymph nodes and bone marrow. J. Exp. Med..

[84] Gregoire C (2007). The trafficking of natural killer cells. Immunol. Rev..

[85] Cyster JG (2005). Chemokines, sphingosine-1-phosphate, and cell migration in secondary lymphoid organs. Annu. Rev. Immunol..

[86] Mehling M (2015). Tissue distribution dynamics of human NK cells inferred from peripheral blood depletion kinetics after sphingosine-1-phosphate receptor blockade. Scand. J. Immunol..

[87] Paust S (2010). Critical role for the chemokine receptor CXCR6 in NK cell-mediated antigen-specific memory of haptens and viruses. Nat. Immunol..

[88] Zhao J (2020). Single-cell RNA sequencing reveals the heterogeneity of liver-resident immune cells in human. Cell Discov..

[89] Chea S (2015). CXCR6 expression is important for retention and circulation of ILC precursors. Mediators Inflamm..

[90] Cuff AO, Perchet T, Dertschnig S, Golub R, Male V (2020). Tbet promotes CXCR6 expression in immature natural killer cells and natural killer cell egress from the bone marrow. Immunology.

[91] Gullberg D (1992). Analysis of alpha 1 beta 1, alpha 2 beta 1 and alpha 3 beta 1 integrins in cell-collagen interactions: identification of conformation dependent alpha 1 beta 1 binding sites in collagen type I. EMBO J..

[92] Kern A, Eble J, Golbik R, Kuhn K (1993). Interaction of type IV collagen with the isolated integrins alpha 1 beta 1 and alpha 2 beta 1. Eur. J. Biochem..

[93] Vandenberg P (1991). Characterization of a type IV collagen major cell binding site with affinity to the alpha 1 beta 1 and the alpha 2 beta 1 integrins. J. Cell Biol..

[94] Fogler WE (1996). NK cell infiltration into lung, liver, and subcutaneous B16 melanoma is mediated by VCAM-1/VLA-4 interaction. J. Immunol..

[95] Hudspeth K (2016). Human liver-resident CD56(bright)/CD16(neg) NK cells are retained within hepatic sinusoids via the engagement of CCR5 and CXCR6 pathways. J. Autoimmun..

[96] Angelo LS, Bimler LH, Nikzad R, Aviles-Padilla K, Paust S (2019). CXCR6(+) NK cells in human fetal liver and spleen possess unique phenotypic and functional capabilities. Front. Immunol..

[97] Cuff AO (2016). Eomeshi NK cells in human liver are long-lived and do not recirculate but can be replenished from the circulation. J. Immunol..

[98] Heydtmann M (2005). CXC chemokine ligand 16 promotes integrin-mediated adhesion of liver-infiltrating lymphocytes to cholangiocytes and hepatocytes within the inflamed human liver. J. Immunol..

[99] Hemmatazad H, Berger MD (2021). CCR5 is a potential therapeutic target for cancer. Expert Opin. Ther. Targets.

[100] Levy ER, Clara JA, Reger RN, Allan DSJ, Childs RW (2021). RNA-seq analysis reveals CCR5 as a key target for CRISPR gene editing to regulate in vivo nk cell trafficking. Cancers (Basel).

[101] Chen Y, Tian Z (2021). Innate lymphocytes: pathogenesis and therapeutic targets of liver diseases and cancer. Cell Mol. Immunol..

[102] Russell E, Conroy MJ, Barr MP (2022). Harnessing natural killer cells in non-small cell lung cancer. Cells.

[103] Marquardt N (2019). Unique transcriptional and protein-expression signature in human lung tissue-resident NK cells. Nat. Commun..

[104] Ferrero MR, Tavares LP, Garcia CC (2021). The dual role of CCR5 in the course of influenza infection: exploring treatment opportunities. Front. Immunol..

[105] Brownlie D (2022). Comparison of lung-homing receptor expression and activation profiles on NK cell and T cell subsets in COVID-19 and influenza. Front. Immunol..

[106] Freud AG (2006). Evidence for discrete stages of human natural killer cell differentiation in vivo. J. Exp. Med..

[107] Lee BJ, Mace EM (2020). From stem cell to immune effector: how adhesion, migration, and polarity shape T-cell and natural killer cell lymphocyte development in vitro and in vivo. Mol. Biol. Cell.

[108] Scoville SD (2016). A progenitor cell expressing transcription factor RORgammat generates all human innate lymphoid cell subsets. Immunity.

[109] Freud AG (2005). A human CD34(+) subset resides in lymph nodes and differentiates into CD56bright natural killer cells. Immunity.

[110] Hase K (2006). The membrane-bound chemokine CXCL16 expressed on follicle-associated epithelium and M cells mediates lympho-epithelial interaction in GALT. J. Immunol..

[111] Wein AN (2019). CXCR6 regulates localization of tissue-resident memory CD8 T cells to the airways. J. Exp. Med..

[112] Fuchs A (2013). Intraepithelial type 1 innate lymphoid cells are a unique subset of IL-12- and IL-15-responsive IFN-gamma-producing cells. Immunity.

[113] Zhang X, Wei H (2021). Role of decidual natural killer cells in human pregnancy and related pregnancy complications. Front. Immunol..

[114] Hanna J (2003). CXCL12 expression by invasive trophoblasts induces the specific migration of CD16- human natural killer cells. Blood.

[115] Carlino C (2008). Recruitment of circulating NK cells through decidual tissues: a possible mechanism controlling NK cell accumulation in the uterus during early pregnancy. Blood.

[116] Carlino C (2012). Chemerin regulates NK cell accumulation and endothelial cell morphogenesis in the decidua during early pregnancy. J. Clin. Endocrinol. Metab..

[117] Sentman CL, Meadows SK, Wira CR, Eriksson M (2004). Recruitment of uterine NK cells: induction of CXC chemokine ligands 10 and 11 in human endometrium by estradiol and progesterone. J. Immunol..

[118] Li ZY (2014). IFN-gamma induces aberrant CD49b(+) NK cell recruitment through regulating CX3CL1: a novel mechanism by which IFN-gamma provokes pregnancy failure. Cell Death Dis..

[119] Powell DW, Pinchuk IV, Saada JI, Chen X, Mifflin RC (2011). Mesenchymal cells of the intestinal lamina propria. Annu. Rev. Physiol..

[120] Sagebiel AF (2019). Tissue-resident Eomes(+) NK cells are the major innate lymphoid cell population in human infant intestine. Nat. Commun..

[121] Cortez VS (2016). Transforming growth factor-beta signaling guides the differentiation of innate lymphoid cells in salivary glands. Immunity.

[122] Dogra P (2020). Tissue determinants of human NK cell development, function, and residence. Cell.

[123] Kunkel EJ (2000). Lymphocyte CC chemokine receptor 9 and epithelial thymus-expressed chemokine (TECK) expression distinguish the small intestinal immune compartment: Epithelial expression of tissue-specific chemokines as an organizing principle in regional immunity. J. Exp. Med.

[124] Campbell DJ, Butcher EC (2002). Rapid acquisition of tissue-specific homing phenotypes by CD4(+) T cells activated in cutaneous or mucosal lymphoid tissues. J. Exp. Med..

[125] Svensson M (2002). CCL25 mediates the localization of recently activated CD8alphabeta(+) lymphocytes to the small-intestinal mucosa. J. Clin. Invest..

[126] Berahovich RD, Lai NL, Wei Z, Lanier LL, Schall TJ (2006). Evidence for NK cell subsets based on chemokine receptor expression. J. Immunol..

[127] Bajenoff M (2006). Natural killer cell behavior in lymph nodes revealed by static and real-time imaging. J. Exp. Med..

[128] Chen S, Kawashima H, Lowe JB, Lanier LL, Fukuda M (2005). Suppression of tumor formation in lymph nodes by L-selectin-mediated natural killer cell recruitment. J. Exp. Med.

[129] Bar-Ephraim YE (2019). CD62L is a functional and phenotypic marker for circulating innate lymphoid cell precursors. J. Immunol..

[130] Frey M (1998). Differential expression and function of L-selectin on CD56bright and CD56dim natural killer cell subsets. J. Immunol..

[131] Pesce S, Moretta L, Moretta A, Marcenaro E (2016). Human NK cell subsets redistribution in pathological conditions: a role for CCR7 receptor. Front. Immunol..

[132] Dutton EE (2019). Peripheral lymph nodes contain migratory and resident innate lymphoid cell populations. Sci. Immunol..

[133] Lu T (2021). Hijacking TYRO3 from tumor cells via trogocytosis enhances nk-cell effector functions and proliferation. Cancer Immunol. Res..

[134] Somanchi SS, Somanchi A, Cooper LJ, Lee DA (2012). Engineering lymph node homing of ex vivo-expanded human natural killer cells via trogocytosis of the chemokine receptor CCR7. Blood.

[135] Fang V (2017). Gradients of the signaling lipid S1P in lymph nodes position natural killer cells and regulate their interferon-gamma response. Nat. Immunol..

[136] Castriconi R (2018). Molecular mechanisms directing migration and retention of natural killer cells in human tissues. Front. Immunol..

[137] Russick J, Torset C, Hemery E, Cremer I (2020). NK cells in the tumor microenvironment: prognostic and theranostic impact. Recent advances and trends. Semin. Immunol..

[138] Cozar B (2021). Tumor-infiltrating natural killer cells. Cancer Discov..

[139] Wu M, Mei F, Liu W, Jiang J (2020). Comprehensive characterization of tumor infiltrating natural killer cells and clinical significance in hepatocellular carcinoma based on gene expression profiles. Biomed. Pharmacother..

[140] Cursons J (2019). A gene signature predicting natural killer cell infiltration and improved survival in melanoma patients. Cancer Immunol. Res..

[141] Barry KC (2018). A natural killer-dendritic cell axis defines checkpoint therapy-responsive tumor microenvironments. Nat. Med..

[142] Soo RA (2018). Prognostic significance of immune cells in non-small cell lung cancer: meta-analysis. Oncotarget.

[143] Villegas FR (2002). Prognostic significance of tumor infiltrating natural killer cells subset CD57 in patients with squamous cell lung cancer. Lung Cancer.

[144] Takanami I, Takeuchi K, Giga M (2001). The prognostic value of natural killer cell infiltration in resected pulmonary adenocarcinoma. J. Thorac. Cardiovasc. Surg..

[145] Remark R (2013). Characteristics and clinical impacts of the immune environments in colorectal and renal cell carcinoma lung metastases: influence of tumor origin. Clin. Cancer Res..

[146] Ishigami S (2000). Prognostic value of intratumoral natural killer cells in gastric carcinoma. Cancer.

[147] Li B, Jiang Y, Li G, Fisher GA, Li R (2020). Natural killer cell and stroma abundance are independently prognostic and predict gastric cancer chemotherapy benefit. JCI Insight.

[148] Lee H (2019). Integrated molecular and immunophenotypic analysis of NK cells in anti-PD-1 treated metastatic melanoma patients. Oncoimmunology.

[149] Gil M, Kim KE (2019). Interleukin-18 is a prognostic biomarker correlated with CD8(+) T cell and natural killer cell infiltration in skin cutaneous melanoma. J. Clin. Med.

[150] Edsparr K, Basse PH, Goldfarb RH, Albertsson P (2011). Matrix metalloproteinases in cytotoxic lymphocytes impact on tumour infiltration and immunomodulation. Cancer Microenviron..

[151] Cichocki F (2020). iPSC-derived NK cells maintain high cytotoxicity and enhance in vivo tumor control in concert with T cells and anti-PD-1 therapy. Sci. Transl. Med..

[152] Moreira A (2019). Adaptive features of natural killer cells in multiple sclerosis. Front. Immunol..

[153] Burke JD, Young HA (2019). IFN-gamma: a cytokine at the right time, is in the right place. Semin. Immunol..

[154] Kaur K (2018). Natural killer cells target and differentiate cancer stem-like cells/undifferentiated tumors: strategies to optimize their growth and expansion for effective cancer immunotherapy. Curr. Opin. Immunol..

[155] Bald T, Krummel MF, Smyth MJ, Barry KC (2020). The NK cell-cancer cycle: advances and new challenges in NK cell-based immunotherapies. Nat. Immunol..

[156] Bottcher JP (2018). NK cells stimulate recruitment of cDC1 into the tumor microenvironment promoting cancer immune control. Cell.

[157] Li Z (2022). ILC1s control leukemia stem cell fate and limit development of AML. Nat. Immunol..

[158] Dong W (2019). The mechanism of anti-PD-L1 antibody efficacy against PD-L1-negative tumors identifies NK cells expressing PD-L1 as a cytolytic effector. Cancer Discov..

[159] Benson DM (2010). The PD-1/PD-L1 axis modulates the natural killer cell versus multiple myeloma effect: a therapeutic target for CT-011, a novel monoclonal anti-PD-1 antibody. Blood.

[160] Gonzalez VD (2021). High-grade serous ovarian tumor cells modulate NK cell function to create an immune-tolerant microenvironment. Cell Rep..

[161] Bassani B (2019). Natural killer cells as key players of tumor progression and angiogenesis: old and novel tools to divert their pro-tumor activities into potent anti-tumor effects. Cancers (Basel).

[162] Chan IS (2020). Cancer cells educate natural killer cells to a metastasis-promoting cell state. J. Cell Biol..

[163] Levi I (2015). Characterization of tumor infiltrating natural killer cell subset. Oncotarget.

[164] Guan Y (2020). Renal cell tumors convert natural killer cells to a proangiogenic phenotype. Oncotarget.

[165] Bruno A (2013). The proangiogenic phenotype of natural killer cells in patients with non-small cell lung cancer. Neoplasia.

[166] Gallazzi M (2020). Prostate cancer peripheral Blood NK cells show enhanced CD9, CD49a, CXCR4, CXCL8, MMP-9 production and secrete monocyte-recruiting and polarizing factors. Front. Immunol..

[167] Cerdeira AS (2013). Conversion of peripheral blood NK cells to a decidual NK-like phenotype by a cocktail of defined factors. J. Immunol..

[168] Bierie B, Moses HL (2006). Tumour microenvironment: TGFbeta: the molecular Jekyll and Hyde of cancer. Nat. Rev. Cancer.

[169] Gotthardt D (2016). STAT5 is a key regulator in nk cells and acts as a molecular switch from tumor surveillance to tumor promotion. Cancer Discov..

[170] Krzywinska E (2017). Loss of HIF-1alpha in natural killer cells inhibits tumour growth by stimulating non-productive angiogenesis. Nat. Commun..

[171] Llovet JM (2022). Immunotherapies for hepatocellular carcinoma. Nat. Rev. Clin. Oncol..

[172] Kumar N, Mroz A, Aletrari M, Goldin R, Purbhoo M (2014). PTU-121Llt1 is upregulated in hepatocellular carcinoma and inhibits Nk cell cytotoxicity. Gut.

[173] Pineiro Fernandez J, Luddy KA, Harmon C, O’Farrelly C (2019). Hepatic tumor microenvironments and effects on NK Cell phenotype and function. Int. J. Mol. Sci..

[174] Li J (2013). The combination of an oxygen-dependent degradation domain-regulated adenovirus expressing the chemokine RANTES/CCL5 and NK-92 cells exerts enhanced antitumor activity in hepatocellular carcinoma. Oncol. Rep..

[175] Nishina S (2019). Dipeptidyl peptidase 4 inhibitors reduce hepatocellular carcinoma by activating lymphocyte chemotaxis in mice. Cell Mol. Gastroenterol. Hepatol..

[176] Bugide S, Gupta R, Green MR, Wajapeyee N (2021). EZH2 inhibits NK cell-mediated antitumor immunity by suppressing CXCL10 expression in an HDAC10-dependent manner. Proc. Natl Acad. Sci. USA.

[177] Chen EB (2019). The miR-561-5p/CX3CL1 signaling axis regulates pulmonary metastasis in hepatocellular carcinoma involving CX3CR1(+) natural killer cells infiltration. Theranostics.

[178] Mossanen JC (2019). CXCR6 inhibits hepatocarcinogenesis by promoting natural killer T- and CD4(+) T-cell-dependent control of senescence. Gastroenterology.

[179] Sun H (2019). Accumulation of tumor-infiltrating CD49a(+) NK cells correlates with poor prognosis for human hepatocellular carcinoma. Cancer Immunol. Res.

[180] Pietra G (2012). Melanoma cells inhibit natural killer cell function by modulating the expression of activating receptors and cytolytic activity. Cancer Res..

[181] van Vliet AA, Georgoudaki AM, Raimo M, de Gruijl TD, Spanholtz J (2021). Adoptive NK cell therapy: a promising treatment prospect for metastatic melanoma. Cancers (Basel).

[182] Kim J (2018). CXCR3-deficient natural killer cells fail to migrate to B16F10 melanoma cells. Int. Immunopharmacol..

[183] Wendel M, Galani IE, Suri-Payer E, Cerwenka A (2008). Natural killer cell accumulation in tumors is dependent on IFN-gamma and CXCR3 ligands. Cancer Res..

[184] Wennerberg E, Kremer V, Childs R, Lundqvist A (2015). CXCL10-induced migration of adoptively transferred human natural killer cells toward solid tumors causes regression of tumor growth in vivo. Cancer Immunol. Immunother..

[185] Cristiani CM (2019). Accumulation of circulating CCR7(+) natural killer cells marks melanoma evolution and reveals a CCL19-dependent metastatic pathway. Cancer Immunol. Res..

[186] Mgrditchian T (2017). Targeting autophagy inhibits melanoma growth by enhancing NK cells infiltration in a CCL5-dependent manner. Proc. Natl Acad. Sci. USA.

[187] Noman MZ, Paggetti J, Moussay E, Berchem G, Janji B (2018). Driving Natural Killer cells toward the melanoma tumor battlefield: autophagy as a valuable therapeutic target. Oncoimmunology.

[188] Noman MZ, Berchem G, Janji B (2018). Targeting autophagy blocks melanoma growth by bringing natural killer cells to the tumor battlefield. Autophagy.

[189] Voshtani R (2019). Progranulin promotes melanoma progression by inhibiting natural killer cell recruitment to the tumor microenvironment. Cancer Lett..

[190] Bhat H (2020). Arenavirus induced CCL5 expression causes NK cell-mediated melanoma regression. Front. Immunol..

[191] Albanesi C (2009). Chemerin expression marks early psoriatic skin lesions and correlates with plasmacytoid dendritic cell recruitment. J. Exp. Med..

[192] Parolini S (2007). The role of chemerin in the colocalization of NK and dendritic cell subsets into inflamed tissues. Blood.

[193] Pachynski RK (2012). The chemoattractant chemerin suppresses melanoma by recruiting natural killer cell antitumor defenses. J. Exp. Med.

[194] Song Y (2019). Chemerin partly mediates tumor-inhibitory effect of all-trans retinoic acid via CMKLR1-dependent natural killer cell recruitment. Immunology.

[195] Kang JY, Kim KE (2021). Prognostic value of interleukin-32 expression and its correlation with the infiltration of natural killer cells in cutaneous melanoma. J. Clin. Med..

[196] Liu J (2014). Formyl peptide receptor suppresses melanoma development and promotes NK cell migration. Inflammation.

[197] Loibl S, Poortmans P, Morrow M, Denkert C, Curigliano G (2021). Breast cancer. Lancet.

[198] Tallerico R (2017). NK cells control breast cancer and related cancer stem cell hematological spread. Oncoimmunology.

[199] Rezaeifard S, Talei A, Shariat M, Erfani N (2021). Tumor infiltrating NK cell (TINK) subsets and functional molecules in patients with breast cancer. Mol. Immunol..

[200] Bouzidi L (2021). Prognostic value of natural killer cells besides tumor-infiltrating lymphocytes in breast cancer tissues. Clin. Breast Cancer.

[201] Yoon MS (2016). Irradiation of breast cancer cells enhances CXCL16 ligand expression and induces the migration of natural killer cells expressing the CXCR6 receptor. Cytotherapy.

[202] Araujo JM (2018). Effect of CCL5 expression in the recruitment of immune cells in triple negative breast cancer. Sci. Rep..

[203] Kim KS (2020). Cationic nanoparticle-mediated activation of natural killer cells for effective cancer immunotherapy. ACS Appl. Mater. Interfaces.

[204] Kandell WM (2020). MicroRNA-155 governs SHIP-1 expression and localization in NK cells and regulates subsequent infiltration into murine AT3 mammary carcinoma. PLoS ONE.

[205] Yu Y (2016). Mesenchymal stem cells with Sirt1 overexpression suppress breast tumor growth via chemokine-dependent natural killer cells recruitment. Sci. Rep..

[206] Correia AL (2021). Hepatic stellate cells suppress NK cell-sustained breast cancer dormancy. Nature.

[207] Chen Q, He L, Li X, Xu L, Chen T (2022). Ruthenium complexes boost NK cell immunotherapy via sensitizing triple-negative breast cancer and shaping immuno-microenvironment. Biomaterials.

[208] Siegel RL, Miller KD, Fuchs HE, Jemal A (2022). Cancer statistics, 2022. CA Cancer J. Clin..

[209] Marquardt N (2017). Human lung natural killer cells are predominantly comprised of highly differentiated hypofunctional CD69(-)CD56(dim) cells. J. Allergy Clin. Immunol..

[210] Cong J (2018). Dysfunction of natural killer cells by FBP1-induced inhibition of glycolysis during lung cancer progression. Cell Metab..

[211] Russick J (2020). Natural killer cells in the human lung tumor microenvironment display immune inhibitory functions. J. Immunother. Cancer.

[212] Yamamoto Y (2018). Lung-resident natural killer cells control pulmonary tumor growth in mice. Cancer Sci..

[213] Que ZJ (2021). Jinfukang inhibits lung cancer metastasis by upregulating CX3CL1 to recruit NK cells to kill CTCs. J. Ethnopharmacol..

[214] Meraz IM (2018). TUSC2 immunogene therapy synergizes with Anti-PD-1 through enhanced proliferation and infiltration of natural killer cells in syngeneic Kras-Mutant mouse lung cancer models. Cancer Immunol. Res..

[215] Yan X (2022). Rocaglamide promotes the infiltration and antitumor immunity of NK cells by activating cGAS-STING signaling in non-small cell lung cancer. Int. J. Biol. Sci..

[216] Yao C (2018). Rocaglamide enhances NK cell-mediated killing of non-small cell lung cancer cells by inhibiting autophagy. Autophagy.

[217] Strizova Z (2019). NK and T cells with a cytotoxic/migratory phenotype accumulate in peritumoral tissue of patients with clear cell renal carcinoma. Urol. Oncol..

[218] Gudowska-Sawczuk M, Kudelski J, Mroczko B (2020). The role of chemokine receptor CXCR3 and its ligands in renal cell carcinoma. Int. J. Mol. Sci..

[219] Kremer V (2017). Genetic engineering of human NK cells to express CXCR2 improves migration to renal cell carcinoma. J. Immunother. Cancer.

[220] Halama N (2011). Natural killer cells are scarce in colorectal carcinoma tissue despite high levels of chemokines and cytokines. Clin. Cancer Res..

[221] Elemam NM, Al-Jaderi Z, Hachim MY, Maghazachi AA (2019). HCT-116 colorectal cancer cells secrete chemokines which induce chemoattraction and intracellular calcium mobilization in NK92 cells. Cancer Immunol. Immunother..

[222] Degos C (2019). Endometrial tumor microenvironment alters human NK cell recruitment, and resident NK cell phenotype and function. Front. Immunol..

[223] Fitzgerald AA (2021). DPP inhibition alters the CXCR3 axis and enhances NK and CD8+ T cell infiltration to improve anti-PD1 efficacy in murine models of pancreatic ductal adenocarcinoma. J. Immunother. Cancer.

[224] Wang J (2021). Multispecific targeting of glioblastoma with tumor microenvironment-responsive multifunctional engineered NK cells. Proc. Natl Acad. Sci. USA.

[225] Tian L (2022). Targeting Fc receptor-mediated effects and the “Don’t Eat Me” signal with an oncolytic virus expressing an anti-cd47 antibody to treat metastatic ovarian cancer. Clin. Cancer Res.

[226] Xu B (2021). An oncolytic virus expressing a full-length antibody enhances antitumor innate immune response to glioblastoma. Nat. Commun..

[227] Ma R (2021). An oncolytic virus expressing IL15/IL15ralpha combined with off-the-shelf EGFR-CAR NK cells targets glioblastoma. Cancer Res.

[228] Han J (2015). TGFbeta treatment enhances glioblastoma virotherapy by inhibiting the innate immune response. Cancer Res.

[229] Alvarez-Breckenridge CA (2012). NK cells impede glioblastoma virotherapy through NKp30 and NKp46 natural cytotoxicity receptors. Nat. Med..

[230] Alvarez-Breckenridge CA (2012). The histone deacetylase inhibitor valproic acid lessens NK cell action against oncolytic virus-infected glioblastoma cells by inhibition of STAT5/T-BET signaling and generation of gamma interferon. J. Virol..

[231] Teng KY (2022). Off-the-shelf prostate stem cell antigen-directed chimeric antigen receptor natural killer cell therapy to treat pancreatic cancer. Gastroenterology.

[232] Deng Y (2015). Transcription factor Foxo1 is a negative regulator of natural killer cell maturation and function. Immunity.

[233] Wang Y (2018). SMAD4 promotes TGF-beta-independent NK cell homeostasis and maturation and antitumor immunity. J. Clin. Invest..

[234] Wang Y (2019). The IL-15-AKT-XBP1s signaling pathway contributes to effector functions and survival in human NK cells. Nat. Immunol..

[235] Townsend MJ (2004). T-bet regulates the terminal maturation and homeostasis of NK and Valpha14i NKT cells. Immunity.

[236] Yu J (2006). Pro- and antiinflammatory cytokine signaling: reciprocal antagonism regulates interferon-gamma production by human natural killer cells. Immunity.

[237] Sun H (2019). Human CD96 correlates to natural killer cell exhaustion and predicts the prognosis of human hepatocellular carcinoma. Hepatology.

[238] Holmes ML (2014). Peripheral natural killer cell maturation depends on the transcription factor Aiolos. EMBO J..

[239] Samson SI (2003). GATA-3 promotes maturation, IFN-gamma production, and liver-specific homing of NK cells. Immunity.

[240] Hess LU (2020). The transcription factor promyelocytic leukemia zinc finger protein is associated with expression of liver-homing receptors on human blood CD56(bright) natural killer cells. Hepatol. Commun..

[241] Kared H (2018). Adaptive NKG2C(+)CD57(+) natural killer cell and Tim-3 expression during viral infections. Front. Immunol..

[242] Schuch A (2019). NK-cell responses are biased towards CD16-mediated effector functions in chronic hepatitis B virus infection. J. Hepatol..

[243] Ma S (2021). The RNA m6A reader YTHDF2 controls NK cell antitumor and antiviral immunity. J. Exp. Med..

[244] Beider K (2003). Involvement of CXCR4 and IL-2 in the homing and retention of human NK and NK T cells to the bone marrow and spleen of NOD/SCID mice. Blood.

[245] Hydes T (2018). IL-12 and IL-15 induce the expression of CXCR6 and CD49a on peripheral natural killer cells. Immun. Inflamm. Dis..

[246] Hodge DL (2002). IL-2 and IL-12 alter NK cell responsiveness to IFN-gamma-inducible protein 10 by down-regulating CXCR3 expression. J. Immunol..

[247] Lusty E (2017). IL-18/IL-15/IL-12 synergy induces elevated and prolonged IFN-gamma production by ex vivo expanded NK cells which is not due to enhanced STAT4 activation. Mol. Immunol..

[248] Allavena P (1994). Interleukin-12 is chemotactic for natural killer cells and stimulates their interaction with vascular endothelium. Blood.

[249] Carson WE (1994). Interleukin (IL) 15 is a novel cytokine that activates human natural killer cells via components of the IL-2 receptor. J. Exp. Med.

[250] Barlic J, Sechler JM, Murphy PM (2003). IL-15 and IL-2 oppositely regulate expression of the chemokine receptor CX3CR1. Blood.

[251] Sechler JM, Barlic J, Grivel JC, Murphy PM (2004). IL-15 alters expression and function of the chemokine receptor CX3CR1 in human NK cells. Cell Immunol..

[252] Moustaki A, Argyropoulos KV, Baxevanis CN, Papamichail M, Perez SA (2011). Effect of the simultaneous administration of glucocorticoids and IL-15 on human NK cell phenotype, proliferation and function. Cancer Immunol. Immunother..

[253] Fu Q (2019). CD83(+) CCR7(+) NK cells induced by interleukin 18 by dendritic cells promote experimental autoimmune uveitis. J. Cell Mol. Med..

[254] Mailliard RB (2005). IL-18-induced CD83+CCR7+ NK helper cells. J. Exp. Med..

[255] Pak-Wittel MA, Yang L, Sojka DK, Rivenbark JG, Yokoyama WM (2013). Interferon-gamma mediates chemokine-dependent recruitment of natural killer cells during viral infection. Proc. Natl Acad. Sci. USA.

[256] Yu XX (2018). Effect of the in vivo application of granulocyte colony-stimulating factor on NK cells in bone marrow and peripheral blood. J. Cell Mol. Med..

[257] Castriconi R (2013). Neuroblastoma-derived TGF-beta1 modulates the chemokine receptor repertoire of human resting NK cells. J. Immunol..

[258] Shaim H (2021). Targeting the alphav integrin/TGF-beta axis improves natural killer cell function against glioblastoma stem cells. J. Clin. Invest.

[259] Parodi M (2018). Hypoxia modifies the transcriptome of human NK cells, modulates their immunoregulatory profile, and influences NK cell subset migration. Front. Immunol..

[260] Husain Z, Huang Y, Seth P, Sukhatme VP (2013). Tumor-derived lactate modifies antitumor immune response: effect on myeloid-derived suppressor cells and NK cells. J. Immunol..

[261] Brand A (2016). LDHA-associated lactic acid production blunts tumor immunosurveillance by T and NK cells. Cell Metab..

[262] Ohta A (2016). A metabolic immune checkpoint: adenosine in tumor microenvironment. Front. Immunol..

[263] Young A (2018). A2AR adenosine signaling suppresses natural killer cell maturation in the tumor microenvironment. Cancer Res..

[264] Jacobs B (2021). Characterization and manipulation of the crosstalk between dendritic and natural killer cells within the tumor microenvironment. Front. Immunol..

[265] Mittal D (2017). Interleukin-12 from CD103(+) Batf3-dependent dendritic cells required for nk-cell suppression of metastasis. Cancer Immunol. Res..

[266] Anguille S (2015). Interleukin-15 dendritic cells harness NK cell cytotoxic effector function in a contact- and IL-15-dependent manner. PLoS ONE.

[267] Semino C, Angelini G, Poggi A, Rubartelli A (2005). NK/iDC interaction results in IL-18 secretion by DCs at the synaptic cleft followed by NK cell activation and release of the DC maturation factor HMGB1. Blood.

[268] Spranger S, Dai D, Horton B, Gajewski TF (2017). Tumor-residing Batf3 dendritic cells are required for effector T cell trafficking and adoptive T cell therapy. Cancer Cell.

[269] Persson CM, Chambers BJ (2010). Plasmacytoid dendritic cell-induced migration and activation of NK cells in vivo. Eur. J. Immunol..

[270] Doorduijn EM (2017). CD4(+) T cell and NK cell interplay key to regression of MHC class I(low) tumors upon TLR7/8 agonist therapy. Cancer Immunol. Res..

[271] Bai L (2019). CD8(+) T cells promote maturation of liver-resident NK cells through the CD70-CD27 axis. Hepatology.

[272] Rascle P (2021). NK-B cell cross talk induces CXCR5 expression on natural killer cells. iScience.

[273] Hoegl S (2017). NK cells regulate CXCR2+ neutrophil recruitment during acute lung injury. J. Leukoc. Biol..

[274] Benigni G (2017). CXCR3/CXCL10 axis regulates neutrophil-NK cell cross-talk determining the severity of experimental osteoarthritis. J. Immunol..

[275] Luginbuehl V, Abraham E, Kovar K, Flaaten R, Müller AMS (2022). Better by design: What to expect from novel CAR-engineered cell therapies?. Biotechnol. Adv..

[276] Pan K (2022). CAR race to cancer immunotherapy: from CAR T, CAR NK to CAR macrophage therapy. J. Exp. Clin. Cancer Res..

[277] Ruppel KE, Fricke S, Köhl U, Schmiedel D (2022). Taking lessons from CAR-T cells and going beyond: tailoring design and signaling for CAR-NK Cells in cancer therapy. Front. Immunol..

[278] Zhang C, Hu Y, Xiao W, Tian Z (2021). Chimeric antigen receptor- and natural killer cell receptor-engineered innate killer cells in cancer immunotherapy. Cell Mol. Immunol..

[279] Chen Y (2021). CAR-macrophage: a new immunotherapy candidate against solid tumors. Biomed. Pharmacother..

[280] Cortés-Selva D, Dasgupta B, Singh S, Grewal IS (2021). Innate and innate-like cells: the future of chimeric antigen receptor (car) cell therapy. Trends Pharm. Sci..

[281] Sebestyen Z, Prinz I, Dechanet-Merville J, Silva-Santos B, Kuball J (2020). Translating gammadelta (gammadelta) T cells and their receptors into cancer cell therapies. Nat. Rev. Drug Discov..

[282] Hartmann J, Schussler-Lenz M, Bondanza A, Buchholz CJ (2017). Clinical development of CAR T cells-challenges and opportunities in translating innovative treatment concepts. EMBO Mol. Med.

[283] Gardner RA (2017). Intent-to-treat leukemia remission by CD19 CAR T cells of defined formulation and dose in children and young adults. Blood.

[284] Raje N (2019). Anti-BCMA CAR T-Cell Therapy bb2121 in relapsed or refractory multiple myeloma. N. Engl. J. Med..

[285] Wrona E, Borowiec M, Potemski P (2021). CAR-NK cells in the treatment of solid tumors. Int. J. Mol. Sci..

[286] Lu T (2022). Off-the-shelf CAR natural killer cells secreting IL-15 target spike in treating COVID-19. Nat. Commun..

[287] Ivashkiv LB (2018). IFNgamma: signalling, epigenetics and roles in immunity, metabolism, disease and cancer immunotherapy. Nat. Rev. Immunol..

[288] Guerra E, Di Pietro R, Basile M, Trerotola M, Alberti S (2021). Cancer-homing CAR-T cells and endogenous immune population dynamics. Int. J. Mol. Sci..

[289] Liu G (2020). CXCR2-modified CAR-T cells have enhanced trafficking ability that improves treatment of hepatocellular carcinoma. Eur. J. Immunol..

[290] Li G (2021). IL-7 and CCR2b co-expression-mediated enhanced CAR-T survival and infiltration in solid tumors. Front. Oncol..

[291] Li G (2021). CXCR5 guides migration and tumor eradication of anti-EGFR chimeric antigen receptor T cells. Mol. Ther. Oncolytics.

[292] Ng YY, Tay JCK, Wang S (2020). CXCR1 expression to improve anti-cancer efficacy of intravenously injected CAR-NK cells in mice with peritoneal xenografts. Mol. Ther. Oncolytics.

[293] Muller N (2015). Engineering NK cells modified with an EGFRvIII-specific chimeric antigen receptor to overexpress cxcr4 improves immunotherapy of CXCL12/SDF-1alpha-secreting glioblastoma. J. Immunother..

[294] Carlsten M (2016). Efficient mRNA-based genetic engineering of human NK cells with high-affinity CD16 and CCR7 augments rituximab-induced ADCC against lymphoma and targets NK cell migration toward the lymph node-associated chemokine CCL19. Front. Immunol..

[295] Liu E (2018). Cord blood NK cells engineered to express IL-15 and a CD19-targeted CAR show long-term persistence and potent antitumor activity. Leukemia.

[296] Du Z, Ng YY, Zha S, Wang S (2021). piggyBac system to co-express NKG2D CAR and IL-15 to augment the in vivo persistence and anti-AML activity of human peripheral blood NK cells. Mol. Ther. Methods Clin. Dev..

[297] Zheng X (2022). The use of supercytokines, immunocytokines, engager cytokines, and other synthetic cytokines in immunotherapy. Cell Mol. Immunol..

[298] Wang Z (2017). Augmented anti-tumor activity of NK-92 cells expressing chimeric receptors of TGF-betaR II and NKG2D. Cancer Immunol. Immunother..

[299] Mohammed S (2017). Improving chimeric antigen receptor-modified T cell function by reversing the immunosuppressive tumor microenvironment of pancreatic cancer. Mol. Ther..

[300] Morty RE (2020). Using experimental models to identify pathogenic pathways and putative disease management targets in bronchopulmonary dysplasia. Neonatology.

[301] Parihar R (2019). NK cells expressing a chimeric activating receptor eliminate MDSCs and rescue impaired CAR-T cell activity against solid tumors. Cancer Immunol. Res..

[302] Rosenberg SA (1987). Autologous bone marrow transplantation in non-Hodgkin’s lymphoma. N. Engl. J. Med..

[303] Escudier B (1993). Combination of interleukin-2 and gamma interferon in metastatic renal cell carcinoma. Eur. J. Cancer.

[304] Ferrini S (1992). Targeting of T or NK lymphocytes against tumor cells by bispecific monoclonal antibodies: role of different triggering molecules. Int. J. Cancer Suppl..

[305] Kohrt HE (2014). Anti-KIR antibody enhancement of anti-lymphoma activity of natural killer cells as monotherapy and in combination with anti-CD20 antibodies. Blood.

[306] Vallera DA (2016). IL15 trispecific killer engagers (TriKE) make natural killer cells specific to CD33+ targets while also inducing persistence, in vivo expansion, and enhanced function. Clin. Cancer Res.

[307] Iliopoulou EG (2010). A phase I trial of adoptive transfer of allogeneic natural killer cells in patients with advanced non-small cell lung cancer. Cancer Immunol. Immunother..

[308] Dolstra H (2017). Successful transfer of umbilical cord blood CD34(+) hematopoietic stem and progenitor-derived NK cells in older acute myeloid leukemia patients. Clin. Cancer Res..

[309] Moreno L (2019). The mechanism of action of the anti-CD38 monoclonal antibody isatuximab in multiple myeloma. Clin. Cancer Res..

[310] Albinger N (2022). Primary CD33-targeting CAR-NK cells for the treatment of acute myeloid leukemia. Blood Cancer J..

